# Microwave-to-optical transduction with erbium ions coupled to planar photonic and superconducting resonators

**DOI:** 10.1038/s41467-023-36799-0

**Published:** 2023-03-01

**Authors:** Jake Rochman, Tian Xie, John G. Bartholomew, K. C. Schwab, Andrei Faraon

**Affiliations:** 1 Kavli Nanoscience Institute and Thomas J. Watson, Sr., Laboratory of Applied Physics, California Institute of Technology, Pasadena, CA 91125 USA; 2 Institute for Quantum Information and Matter, California Institute of Technology, Pasadena, CA 91125 USA; 3 The University of Sydney Nano Institute, The University of Sydney, Sydney, NSW 2006 Australia; 4 Present Address: Centre for Engineered Quantum Systems, School of Physics, The University of Sydney, Sydney, NSW 2006 Australia

**Keywords:** Quantum optics, Nanophotonics and plasmonics, Magneto-optics, Single photons and quantum effects

## Abstract

Optical quantum networks can connect distant quantum processors to enable secure quantum communication and distributed quantum computing. Superconducting qubits are a leading technology for quantum information processing but cannot couple to long-distance optical networks without an efficient, coherent, and low noise interface between microwave and optical photons. Here, we demonstrate a microwave-to-optical transducer using an ensemble of erbium ions that is simultaneously coupled to a superconducting microwave resonator and a nanophotonic optical resonator. The coherent atomic transitions of the ions mediate the frequency conversion from microwave photons to optical photons and using photon counting we observed device conversion efficiency approaching 10^−7^. With pulsed operation at a low duty cycle, the device maintained a spin temperature below 100 mK and microwave resonator heating of less than 0.15 quanta.

## Abstract (web summary)

Interfacing superconducting quantum information processors with long-distance optical networks would require coherent interfacing between microwave and optical photons. Here, the authors show a chip-integrated microwave-to-optical transducer based on rare earth ion ensembles.

## Introduction

Quantum transducers that convert photons between different energies are essential components of a hybrid quantum network^1,2^. Transducers that operate between microwave and optical frequencies are of particular interest to interface state-of-the-art cryogenic superconducting quantum circuits^3^, with excitations at microwave frequencies, and room temperature optical quantum networks using telecom photons^4^. Efficient, low-noise, and high bandwidth microwave-to-optical quantum transducers can permit superconducting circuits to function within large-scale and long-distance quantum communication and distributed quantum computing systems^5,6^. High-efficiency transduction requires strong coupling between the transduction medium and both optical and microwave photons. Low noise transduction requires low temperature operation (T < $\hslash$ω/k_B_), and minimal decoherence or added parasitic photons from the conversion process. Efforts to develop a microwave-to-optical transducer have focused on schemes using an intermediate mechanical mode^7,8^ or electro-optic materials^9,10^, while other approaches, such as atomic ensembles^11–13^ or magnonic systems^14^, have also been demonstrated recently.

**Fig. 1:**
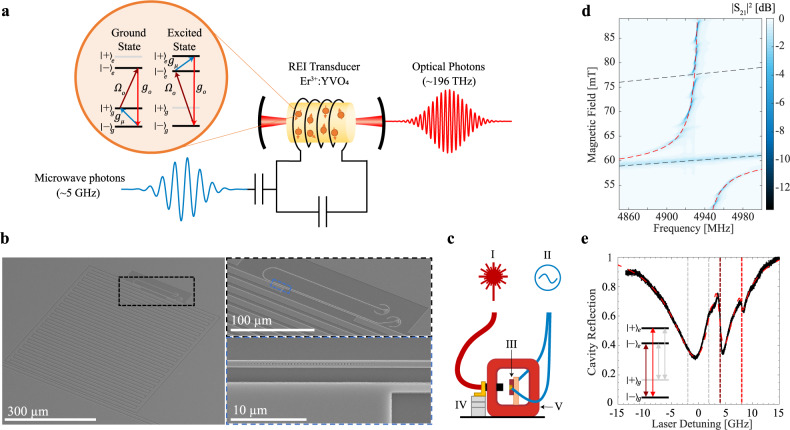
Integrated rare-earth ion transducer and ion-cavity coupling characterization. **a** Schematic of the rare-earth ion transducer using Er^3+^:YVO_4_. The erbium ions simultaneously couple to an optical cavity and a superconducting microwave cavity. Microwave photons can couple to either the ground state or excited state spin transitions (${{{{{{\rm{g}}}}}}}_{\mu }$), while optical photons can couple to four different optical transitions (${\Omega }_{o}$ for the pump and ${g}_{o}$ for the output photon). The two levels in the ground state are labeled ${|-\rangle }_{g}\,\&\,{ |+\rangle }_{g}$ and the two levels in the excited state are labeled ${|-\rangle }_{e}\,\&\,{ |+\rangle }_{e}$, where the+,- corresponds to the sign of the crystal field quantum number of the states. Transduction is achieved by mixing a microwave photon with an optical pump field and generating an optical photon, where each light field couples to an erbium transition. **b** SEM images of the rare-earth ion transducer. The full device including the microwave resonator and optical resonator on the Er^3+^:YVO_4_ substrate are shown on the left, while the zoomed images show a close up of the optical resonator next to the inductive wire and a photonic crystal mirror, respectively. **c** Schematic of the transducer characterization set-up in the dilution refrigerator that enables optical (I) and microwave (II) coupling to the device (III) and static magnetic field control with a superconducting magnet (V). The optical fiber input was on a piezo stage (IV) for alignment. **d** Microwave transmission spectrum showing the coupling between the erbium ground state spins and the superconducting microwave cavity as a function of the applied magnetic field. The simulation fit shown by the dashed red line follows the model described in Supplementary Note 1. The ground (excited) state spin transition is represented with the lower (upper) dashed line. **e** Optical cavity reflection spectrum showing the coupling to the erbium optical transitions with a magnetic field of 76 mT, which is the magnetic field used for excited state transduction. The four vertical lines are a guide for the four optical transitions. Due to the low temperature during the measurement, the population of ${ |+\rangle }_{g}$ was small and coupling to transitions involving this level were suppressed. The simulation fit shown by the dashed red line follows the model described in Supplementary Note 1.

Ensembles of rare-earth ions (REIs) in crystals offer a promising platform for microwave-to-optical transduction (Fig. 1a). Efficient transduction can be achieved by simultaneous strong ensemble coupling of REI’s Zeeman or hyperfine transitions to a microwave resonator and their coherent 4f-4f optical transitions to an optical cavity. Also, REIs offer advantages for operating in the low noise regime due to the absence of a mechanical mode that can be susceptible to thermal excitations and they have narrow atomic transition inhomogeneities (i.e. much smaller than the microwave transduction frequency) that intrinsically minimize the Stokes noise process^15^. Further, REIs systems have demonstrated additional quantum network resources such as quantum memories^16^ and single-photon sources^17,18^, which can enable additional functionality when combined with a REI-based transducer.

Previous REI transducers include bulk crystals with macroscopic resonators that require high optical pump power^11^ or on-chip optical and microwave waveguides with limited efficiency^12^. These implementations have used a Raman scattering protocol that requires a three-level atomic system^19^. The input microwave (optical) field induces coherence of a REI microwave (optical) transition, which is transferred to an optical (microwave) coherence using a parametric optical pump field. An integrated REI transducer platform offers the advantage of smaller optical mode volumes, which permit lower optical power for equivalent pump Rabi frequencies and a path toward high-density integration with superconducting quantum circuits. To improve the efficiency from initial on-chip REI transducers^12^, it is essential to use integrated optical and microwave resonators for increasing the coupling strength between the fields and the REI ensemble.

We report an architecture for REI-based transduction with an integrated superconducting microwave resonator and a photonic crystal optical resonator patterned on a Er^3+^:YVO_4_ substrate. We demonstrate coherent microwave-to-optical transduction from 5 GHz photons to 196 THz photons at 100 mK within a dilution refrigerator. Using single-photon detection, we measured the transducer in a pulsed mode where we observed increased efficiency and lower temperature of the spin system compared to continuous-wave operation. Lastly, we characterized the heating of our system by measuring the spin temperature and microwave resonator noise induced by the incident optical pump. This work represents a significant step toward a useful REI microwave-to-optical quantum transducer.

## Results

### Integrated rare-earth ion transducer

The optical resonator consists of a transverse-magnetic (TM) mode amorphous silicon waveguide (l_o _= 100 µm) between two photonic crystal mirrors. The cavity field evanescently couples to the erbium ions in the YVO_4_ substrate (560 ppm natural abundance doping concentration) (Fig. 1b). The superconducting niobium microwave resonator (resonant frequency ω_µ_ = 2π $\times$4.94 GHz) is composed of a narrow inductive wire (*w*_ind_ = 1 µm, *l*_ind_ = 100 µm) that was patterned next to the optical resonator (gap = 1.2 µm) and is shunted by an interdigitated capacitor. The erbium ions act as a magneto-optic transducer, which requires simultaneous coupling to the microwave magnetic field and the optical resonator field. A niobium co-planar waveguide patterned on the YVO substrate was used to capacitively couple to the microwave resonator, while a grating coupler (~30% efficiency) was used for optical coupling from an optical fiber (Fig. 1c). The device was mounted on the mixing chamber stage of a dilution refrigerator with a base temperature of 35 mK. A two-axis home-built superconducting magnet generated a static in-plane magnetic field with respect to the substrate surface for tuning the erbium spin transitions into resonance with the microwave cavity. Nitrogen gas condensation was used for tuning the optical cavity into resonance with the erbium ^4^I_15/2_-^4^I_13/2_ optical transitions.

Er^3+^:YVO_4_ has been shown to be a promising material for microwave-to-optical transduction due to its relatively strong and narrow optical transitions in bulk crystals and telecom optical transition wavelengths^20^. For our integrated transducer, we used an in-plane magnetic field at an angle of 50^o^ with respect to the crystal c-axis. In this field orientation, magnetic dipole transitions are allowed for all four optical transitions, which can be frequency resolved because the electron-spin g-factor is different for the optical ground and excited states.

We first independently characterized the ion-cavity coupling between the erbium ensemble and the microwave and optical resonators. At microwave frequencies, this was conducted by sweeping the magnetic field to bring the erbium spin transitions through the microwave cavity resonance and measuring the cavity transmission spectrum on a network analyzer (Fig. 1d). At a magnetic field of 60 mT, we observed an avoided-crossing due to the large ensemble coupling between the microwave cavity and the erbium ground state spins (${g}_{\mu,{{{{{\rm{tot}}}}}}}/2\pi$ = 105 MHz) at the base temperature. Using the measured cavity energy decay rate of ${\kappa }_{\mu }/2\pi$ = 2 MHz, and spin inhomogeneous linewidth of ${\Delta }_{\mu }/2\pi$ = 65 MHz, we obtained a microwave ensemble co-operativity of ${C}_{\mu }=4{g}_{\mu,{{{{{\rm{tot}}}}}}}^{2}/{\kappa }_{\mu }{\Delta }_{\mu }$ = 340. We also observed coupling of the microwave resonator to several spin transitions that we attribute to the ^167^Er isotope (see Supplementary Note 9 for details) and coupling of the spins directly with the co-planar waveguide.

The optical ion-cavity coupling was measured by sweeping the frequency of a weak probe laser (${P}_{o}$~1 pW) across the cavity resonance and detecting the light reflected from the cavity on a superconducting nanowire single-photon detector (SNSPD) (Fig. 1e). A magnetic field of 76 mT was applied such that each transition was resolved, and this is the field used later for excited state transduction. Due to the relatively large cavity linewidth (i.e. ${\kappa }_{o} > {\omega }_{\mu }$), all the optical transitions coupled to the same optical cavity mode. From fitting the ion-cavity spectrum^21^, we obtained ${\kappa }_{o}/2\pi$ = 13.2 GHz and ${g}_{o,{{{{{\rm{tot}}}}}},\parallel }/2\pi$ = 2.0 GHz for the optical transition ${|-\rangle }_{g}\leftrightarrow {|-\rangle }_{e}$ and ${g}_{o,{tot},\perp }/2\pi$ = 0.98 GHz for transition ${|-\rangle }_{g}\leftrightarrow { |+\rangle }_{e}$. The inhomogeneous linewidth was measured in photoluminescence (see Supplementary Note 8) to be ${\Delta }_{o}/2\pi$ = 300 MHz resulting in an optical ensemble co-operativity of ${C}_{o,\parallel }$ = 4.1 and ${C}_{o,\perp }$ = 1.1 for the two optical transitions.

Not all the erbium spins that couple to the microwave resonator can be used for transduction because the mode overlap between the optical and microwave modes is $F=9.5\times {10}^{-4}$ and erbium spins are uniformly doped within the substrate. Spins that are only within the microwave cavity act as parasitic spins that can absorb microwave photons but cannot transduce them. The effects from parasitic spins can be suppressed by detuning the spins from the cavity, using the excited state spin for transduction or, for future devices, eliminating their presence by controlling the position of the erbium ions within the device.

### Continuous-wave transduction

**Fig. 2:**
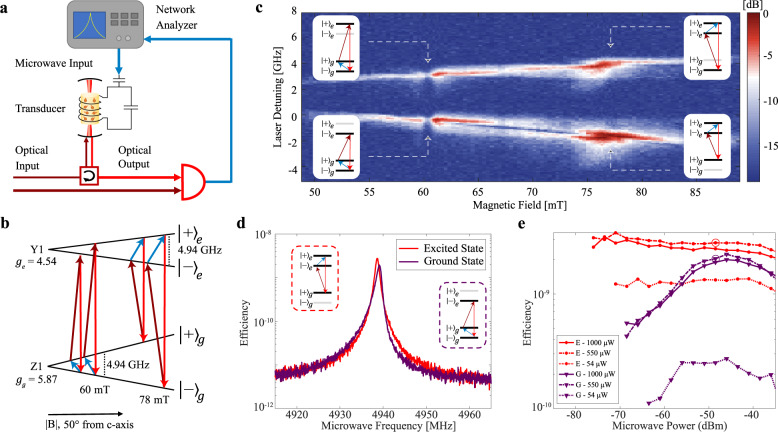
CW M2O transduction efficiency with heterodyne detection. **a** CW heterodyne detection set-up where the upconverted optical tone, generated by mixing an optical pump and the microwave tone in the transducer, was mixed with an optical local oscillator on a photodetector. The microwave beat note signal was then detected on a network analyzer. **b** The Er^3+^:YVO_4_ level structure showing all four configurations for generating transduction signals including two using the ground state spin at B~60 mT and two using the excited state spin at B~78 mT. The g-factors of the ground and excited state are shown for the transduction magnetic field angle of 50 degrees from the c-axis. **c** Normalized heterodyne spectrum where the applied bias magnetic field and the pump laser frequency were swept. The four configurations (as labeled in the inset level structure diagrams) for transduction with Er^3+^:YVO_4_ are identified. The microwave frequency was set to the microwave cavity resonance. In the far-detuned case (i.e. 65-70 mT) the signal was split due to absorption from other optical transitions. **d** Transduction efficiency spectrum for the ground (purple) and excited (red) state spins as a function of the applied microwave frequency for ${P}_{o}$ = 550 µW and ${P}_{\mu }$ = −51 dBm. The level structures used for each trace are shown in the insets and are the same in **e**. **e** Transduction efficiency for the ground state (purple) and the excited state (red) at different input optical and microwave power. The circles correspond to the power parameters used in **d**, where the circle color matches the trace color in **d**.

As a first step, we measured coherent microwave-to-optical conversion in continuous-wave (CW) mode using a Raman heterodyne technique^11^. The input microwave photons were transduced to optical photons which were mixed with a local oscillator (LO) on a photodetector. The generated microwave beat note was measured with a network analyzer (Fig. 2a). We characterized the large parameter space by performing a three-dimensional parameter sweep (magnetic field, pump laser frequency and microwave frequency) and observe four atomic configurations that generate a transduction signal. We fixed the optical and microwave power to be ${P}_{o}$ = 7 µW and ${P}_{\mu }$ = −51 dBm (referenced to the input of the device) and obtained a high SNR (18 dB) heterodyne spectrum.

In Fig. 2c, we fixed the microwave frequency on resonance with the microwave cavity (i.e. the microwave frequency with the highest efficiency) and swept the pump laser frequency and the magnetic field strength. There are four configurations for transduction with two conditions that use the ground state spin ($\Lambda$ systems) at ~60 mT and two conditions that use the excited state spin (V system) at ~78 mT. As the magnetic field changes, the transduction signal follows the laser frequency such that the laser is resonant with the optical transitions. The laser frequency difference between the two $\Lambda$ (V) systems corresponds to the excited (ground) state spin frequency. The transduction signal decreased when the microwave cavity was resonant with the ground state spin (60 mT) due to extra losses from the parasitic ions but was increased 8 dB by detuning the spins ~100 MHz at 62 mT.

In Fig. 2d, we show the device transduction efficiency, ${\eta }_{d}$, for both the ground state and excited state at ${P}_{o}$ = 550 µW and ${P}_{\mu }$ = −51 dBm as a function of the input microwave frequency. We define the device transduction efficiency as the ratio of converted optical photons that propagate into the output optical fiber compared to the microwave photons in the input microwave coupling waveguide. We focus on the transduction configurations where the pump laser is coupled to the ${ |+\rangle }_{g}\leftrightarrow {|-\rangle }_{e}$ transition as these configurations have higher efficiency in CW mode. The pump laser frequency was selected to maximize the efficiency and was near the center of the inhomogeneous line.

The transduction efficiency linewidth followed the microwave cavity linewidth, which is the narrowest bandwidth component of the transducer. The ground state signal had an asymmetric line shape due to the large microwave power that drove the spins during the measurement. The bandwidth of the transduction signal was 1 MHz, which matches the microwave cavity linewidth for each measurement.

Next, we determined the efficiency dependence on both the optical and microwave power for ground and excited state transduction (Fig. 2e). In our integrated REI transducer, the transduction efficiency reached a maximum at ${P}_{o}$ = 550 µW. At higher optical power, the efficiency decreased primarily due to device heating, which limited the spin population difference and caused optical transition saturation. We measured a maximum CW device efficiency of ${\eta }_{d}=2\times {10}^{-9}$ in the ground state (${P}_{o}$ = 550 µW/ ${P}_{\mu }$ = −46 dBm) and ${\eta }_{d}=3\times {10}^{-9}$ in the excited state (${P}_{o}$ = 550 µW/ ${P}_{\mu }$ = −71 dBm). At this optical power, we expect a spin temperature of ~2 K (see SI Fig. S13), but the temperature can be reduced by operating in a pulsed mode.

As the microwave power was reduced (i.e. ${P}_{\mu } < -50\,{{{{{\rm{dBm}}}}}}$), the ground state efficiency decreased as the input microwave tone was no longer saturating the absorption from the parasitic spins that diminished the transducer efficiency. This makes excited state transduction more promising in the low microwave power and low temperature regime for this device.

### Pulsed transduction

**Fig. 3:**
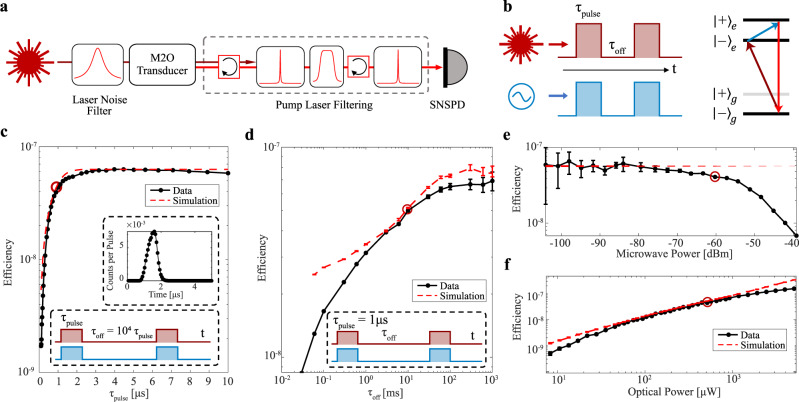
Pulsed excited state transduction efficiency with photon counting detection. **a** Schematic of the pulsed transduction setup using photon counting detection by filtering out the pump laser. Before the transducer one filter was used to remove broadband noise from the laser, while after the transducer the two high finesse Fabry-Perot filters and a set of broadband spectral filters were used to remove the main pump laser tone and broadband noise leakage. **b** Pulse sequence configuration, where we have a square pulse of length, ${\tau }_{{{{{{\rm{pulse}}}}}}}$, and an off time between pulses of length, ${\tau }_{{{{{{\rm{off}}}}}}}$, for both the optical and microwave pulses simultaneously. The level structure used for the excited state transduction process used in c-f. **c**) Pulsed transduction efficiency as a function of the pulse length with a fixed duty cycle ($\frac{{\tau }_{{{{{{\rm{pulse}}}}}}}}{{\tau }_{{{{{{\rm{off}}}}}}}}=0.01\%$). Inset shows the time domain signal of the 1 µs transduction pulse, where we obtain SNR of 40 dB. The simulation plotted in the red dashed line models the pulse spectral overlap with the microwave resonator. **d**–**f** Transduction efficiency as a function of the off time between transduction pulses, the input microwave power, and the input optical pump power, respectively, for a 1 µs transduction pulse. The red circles represent the common experimental condition in the different parameter sweeps. The simulation plotted in the red dashed line follows the model described in Supplementary Note 2. Error bars correspond to $\sqrt{{{{{{\rm{counts}}}}}}}$ measured on the SNSPD and the error bars are propagated to the efficiency. The simulation error bars correspond to the spin temperature uncertainty.

To reduce the device temperature and increase the device efficiency, we characterized the REI transducer operating in pulsed mode. Short transduction pulses and small duty cycles were used to lower the device temperature and minimize optical transition saturation from large driving fields, which limited the device efficiency in CW mode. We also implemented direct photon detection of the transduced optical photons. Here, we attenuated the pump laser photons by ~140 dB with tunable spectral filters and detected the transduced photons using an SNSPD (Fig. 3a). Photon detection is advantageous due to the decreased noise floor, especially for short pulses/ high bandwidth measurements, and for measuring transduced single photons^8^.

For measuring pulsed transduction, we focused on excited state transduction to reduce the effects of the parasitic ions. We applied a magnetic field of 76 mT to avoid the losses associated with the parasitic transitions of the ^167^Er isotope. Future devices can use isotopically purified zero-nuclear-spin erbium isotopes to eliminate this complication. In contrast to the previous CW excited state transduction, we set the optical pump frequency to the ${|-\rangle }_{g}\leftrightarrow { |+\rangle }_{e}$ transition so that the population in the involved ground state increases as the temperature decreases.

We first characterized the transducer bandwidth by measuring the transduced pulse as a function of the pulse duration at a fixed duty cycle (duty cycle = ${\tau }_{{{{{{\rm{pulse}}}}}}}$/${\tau }_{{{{{{\rm{off}}}}}}}$) of 0.01% with ${P}_{\mu }$ = −60 dBm and ${P}_{o}$ = 550 µW (Fig. 3c). We only considered relatively short pulses (${\tau }_{{{{{{\rm{pulse}}}}}}}$ < 10 µs) to avoid significant heating during the transduction pulse. The efficiency reached −3 dB of the maximum when the pulse length decreases to ${\tau }_{{{{{{\rm{pulse}}}}}}}$ = 630 ns, which matches the microwave cavity bandwidth. There is a tradeoff between reducing the average optical power for reduced temperature and the maximum efficiency, so we fixed our transduction pulse length to 1 µs for all subsequent pulsed measurements.

**Fig. 4:**
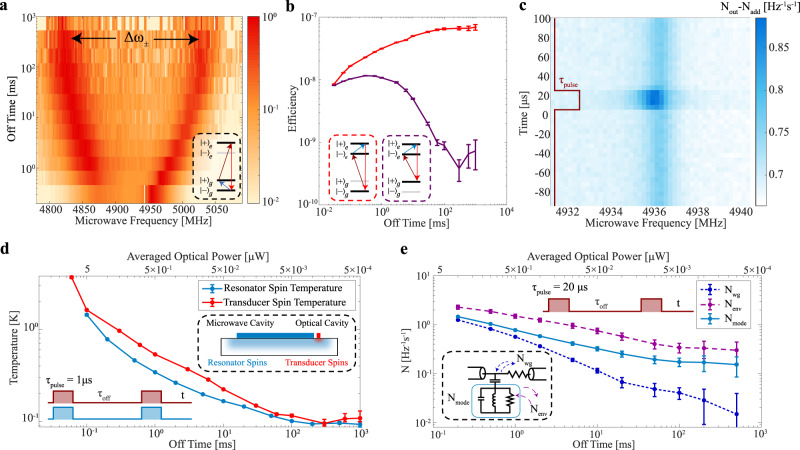
Transducer Temperature Characterization. **a** Pulsed ground state transduction spectrum for different off times between adjacent 1 µs transduction pulses to characterize the temperature of the ground state spins coupled to the microwave resonator. The frequency splitting in the transduction signal ($\Delta { \omega _ \pm }$) matches the microwave resonator mode splitting and indicates the ground state spin population distribution. **b** Pulsed excited state transduction efficiency for coupling to both ground state levels (lower ground state level in red and the higher ground state level in purple) as a function of the off time (${\tau _{{{{{\rm{off}}}}}}}$) between adjacent 1 µs transduction pulses. The efficiency ratio corresponds to the temperature of the ground state spins within our transducer. **c** Time-resolved microwave resonator noise spectrum for an optical pulse sequence with ${P}_{o}$ = 550 µW, ${\tau }_{{{{{{\rm{pulse}}}}}}}$ = 20 µs and ${\tau }_{{{{{{\rm{off}}}}}}}$ = 5 ms. The noise spectrum includes contributions from the microwave resonator environment and the microwave coupling waveguide. **d** Temperature of all the spins coupled to the microwave resonator (using data from **a**) and the spins within transducer (using data from **b**) as measured during the transduction sequence for the different pulse sequence off times. The resonator spins correspond to the erbium ions within the microwave resonator mode (light blue region in inset schematic), while the transducer spins correspond to the erbium ions within the optical resonator mode (red in inset schematic). **e** Thermal noise of the microwave resonator mode (turquoise), microwave resonator environment (purple) and microwave coupling waveguide (blue) induced by the optical pump as a function of the pulse off time. The inset schematic shows the radiative coupling between the different baths that determine the microwave resonator mode noise. The error bars correspond to the 95% confidence interval of the fitting.

Next, we measured the transducer efficiency as a function of the off time between adjacent transduction pulses (Fig. 3d). As we increased the off time from 30 µs to 100 ms, we observe an increase in efficiency from ${\eta }_{d}=9\times {10}^{-9}$ to ${\eta }_{d}=7\times {10}^{-8}$. We attribute this increase in efficiency to a reduction in the device temperature and operating with input fields below saturation of the atomic transitions. The decreased spin temperature increases the number of ions in the V system and increases the efficiency. The efficiency did not increase beyond ${\tau }_{{{{{{\rm{off}}}}}}}=100\,{{{{{\rm{ms}}}}}}$ as the spin temperature saturated beyond this off time (Fig. 4d).

The model used for the simulation is described in Supplementary Note 2 and used the parameters in Table S4. The only free parameters of the simulation are the spin and optical dephasing rates. The temperature used in the model is from the experimentally determined data from Fig. 4d.

We also measured the pulsed transduction efficiency as a function of the input microwave power (Fig. 3e). Ideally, we could characterize the transducer at the single photon level where a quantum transducer would operate, but the modest device and detection efficiency and the finite measurement noise of the setup limited measurements to input microwave pulses with ~10^4^ photons. At ${P}_{\mu }$ = −100 dBm (10^4^ microwave photons per pulse), we observed a maximum pulsed excited state transduction efficiency of ${\eta }_{d}=6\times {10}^{-8}$. There is an efficiency roll-off at ${P}_{\mu } \sim$−55 dBm (~10^9^ photons per pulse) that we attribute to saturation of the microwave transition. The linear approximation of the transduction model, which predicts that the efficiency is independent of the microwave input power, is a good approximation up to the efficiency roll-off power.

For the optical power sweep, we observed a continuous increase in the efficiency to ${\eta }_{d}=1\times {10}^{-7}$ with optical pump power up to ${P}_{o}$ = 5 mW, which differs from the CW operation where the efficiency reached a maximum at ${P}_{o}$ = 550 µW (Fig. 3f). Based on the model described in Supplementary Note 2, the transduction efficiency scales linearly with the pump optical power. The rate of efficiency increase with optical power (i.e. d${\eta }_{d}$/d${P}_{o}$) decreases at the highest optical power in the pulsed mode, which suggests we are approaching the highest efficiency for this device in the pulsed operation. We attribute the simulation and experimental deviation to small changes in the optimal magnetic field and laser frequency as the optical power is changed. All experimental parameters were optimized at ${P}_{o}=$ 550 ${{{{{\rm{\mu }}}}}}{{{{{\rm{W}}}}}}$ (highlighted by a red circle in Fig. 3f) and remained fixed during the power sweep.

### Device temperature analysis

During the transduction process, heating from the optical pump can induce noise photons that can pollute the transducer output field. Ideally, to characterize this noise, we can measure the generated noise photons directly at the transducer output. However, due to our limited device efficiency, some noise sources (i.e. thermal microwave excitations) are heavily suppressed at the transducer output, so we cannot faithfully determine the noise contributions from those sources. Instead, we quantify the temperature or noise of different components of the transducer^22–24^.

Here, we measured the temperature of the erbium ions and the microwave resonator noise to quantify the optical heating effects during the transduction process, which we suspect to be the dominating noise source for our REI transducer. For the erbium spins there are two ensembles that we can characterize; all the erbium ions that couple to the microwave resonator and the erbium ions that are used for transduction (i.e. ions within both the optical and microwave mode volumes).

The average temperature of the ensemble of erbium ground state spins that couple to the microwave cavity was determined by measuring the microwave polariton frequency during transduction (Fig. 4a). Optical heating decreases the population difference between the erbium spins, which decreases the microwave cavity mode pulling that determines the microwave frequency that has the largest transduction signal.

We measured the microwave frequency spectrum of the transduction signal for the ground state spin at a magnetic field of 60 mT, ${P}_{o}$= 550 µW, ${P}_{\mu }$ = −60 dBm, ${\tau }_{{pulse}}$ = 1 µs and swept the off time between the pulses to change the average optical power incident on the device. We observed an increase in the transduction signal splitting, corresponding to a decrease in device temperature, as we increase the off time. An average spin temperature of ~100 mK was reached at an off time of 100 ms for the spins coupled to the microwave resonator (Fig. 4d).

The temperature of the erbium spins within the transducer was estimated by measuring the excited state transduction efficiency for the two configurations that interact with each ground state spin level. In the regime where the ion dynamics evolve linearly, the transduction efficiency scales quadratically as a function of population (${\eta }_{d}\propto {N}^{2}$). By measuring the efficiency ratio between the two configurations, we can deduce the ground state spin population distribution and thus the erbium ground state spin temperature.

We measured the transduction efficiency for both V-systems at a magnetic field of 76 mT and keep the input power and pulse sequence the same as in the previous microwave resonator spin temperature measurement (Fig. 4b). As the off time increases, the transducer spin temperature decreases, which results in the V system involving the ${ |+\rangle }_{g}$ state to have decreased efficiency, while the V system involving the ${|-\rangle }_{g}$ state efficiency increases due to increased population and lower device temperature (Fig. 4d). As the off time increased to 300 ms, we measured an efficiency ratio up to 20 dB which corresponds to a transducer spin temperature of 100 mK. The temperature of the spins within the transducer was measured to be slightly higher than the ensemble of spins coupled to the microwave resonator, which we attribute to closer proximity to the optical heating source.

The thermal noise within the microwave resonator due to optical pulses was determined by measuring the thermal noise that coupled to the co-planar waveguide and propagated through the microwave detection setup^25^. After accounting for the added noise (${N}_{{{{{{\rm{add}}}}}}}$ ~ 6.8 [Hz^−1^s^−1^]) and gain ($G$ ~ 90 dB) of the readout amplification chain, we determine the microwave noise from the device including broadband noise from the microwave waveguide, ${N}_{{{{{{\rm{wg}}}}}}}$, and resonant noise from the microwave resonator environment, ${N}_{{{{{{\rm{env}}}}}}}$, that was induced by the optical pulse (see Supplementary Note 13). The microwave resonator mode noise, ${N}_{{{{{{\rm{mode}}}}}}}$, can be determined by a weighted average between these two noise sources based on their respective coupling to the resonator.

Figure 4c shows the time-resolved noise spectrum for optical pulses with ${P}_{o}$ = 550 µW, ${\tau }_{{{{{{\rm{pulse}}}}}}}$ = 20 µs and ${\tau }_{{{{{{\rm{off}}}}}}}$ = 5 ms. We attribute the microwave device noise during the duration of the optical pulse to a fast quasi-particle bath of the niobium resonator. The resonator environment remains hot compared to the waveguide when no light is incident between optical pulses, which we attribute to a slowly-relaxing bath (potentially Er spins or phonon bath) that couples to the microwave resonator.

Next, we changed the off time between the 20 µs optical pulses in the sequence and determine the thermal noise of the microwave resonator mode, waveguide, and resonator environment from the optical excitation pulse (Fig. 4e). We observed a decrease in the resonator noise from the optical pulse to as low as ${N}_{{{{{{\rm{mode}}}}}}}$ = 0.15$\pm 0.07$ quanta, during the optical excitation pulse, for ${\tau }_{{{{{{\rm{off}}}}}}}$ = 500 ms. We fit the off time dependence of the thermal mode occupancy to a power law function and obtain ${N}_{{{{{{\rm{mode}}}}}}}\, \sim \,{\tau }_{{{{{{\rm{off}}}}}}}^{-0.39}$ for off times <20 ms and ${N}_{{{{{{\rm{mode}}}}}}}\, \sim \,{\tau }_{{{{{{\rm{off}}}}}}}^{-0.16}$ for off times longer than 20 ms.

## Discussion

In conclusion, we demonstrated an integrated REI transducer with operation in a dilution refrigerator and characterized its efficiency for the ground and excited state in both the CW and pulsed regime. With both resonators patterned on the surface, this platform is easily transferable to other rare-earth ion/host systems. We measured a transduction device efficiency up to ${\eta }_{d}=3\times {10}^{-9}$ in CW operation and can increase the efficiency of ${\eta }_{d}=1\times {10}^{-7}$ in pulsed operation when ${P}_{o}$ = 5 mW and ${\eta }_{d}=5\times {10}^{-8}$ when ${P}_{o}$ = 550 µW for ${\tau }_{{{{{{\rm{pulse}}}}}}}$ = 1 µs pulses and ${\tau }_{{{{{{\rm{off}}}}}}}$ = 10 ms. We also characterized the temperature of the erbium spins in our device and the microwave resonator noise due to the optical pump (${P}_{o}$= 550 µW) and obtained regimes where the spin temperature reached 100 mK and the microwave resonator heating was 0.15 quanta. We improved the device efficiency by 6 orders of magnitude compared to previous on-chip REI transducers^12^. Also, we were able to achieve a low operation temperature of the transducer, albeit this required long off times between transduction pulses.

We expect significant efficiency improvement (×10^3^) can be achieved by fabricating optical resonators directly out of rare-earth doped crystals, which will increase the number of ions, the optical Rabi frequency and allow us to remove the parasitic spins. Also, over-coupled optical and microwave cavities will increase the total device efficiency and allow us to apply our optical pump field more efficiently. It will allow us to use less optical power and thus reduce the optical heating effects so shorter off times can be used while remaining in the low temperature regime. A path to near unit efficiency is detailed in Supplementary Note 17.

This transducer device operates at a magnetic field of 60–80 mT to bring the erbium spin transition into resonance with the 4.94 GHz microwave cavity. Interfacing the transducer with a superconducting qubit, which traditionally do not operate within large magnetic fields, would require separating the superconducting qubit chip and the transducer with sufficient magnetic shielding in between, interfacing with superconducting qubits that can operate within large magnetic fields^26^ or using other rare-earth ion transitions that allow for transduction at zero or near zero magnetic field^12^.

With these improvements, integrated REI transducers with high-efficiency and low noise operation could be operated with high repetition rates that will allow us to interface our transducer with non-classical light and superconducting circuits.

## Methods

### Fabrication

The transducer was fabricated on a 500 µm thick a-cut Er^3+^:YVO_4_ substrate (560 ppm natural abundance doping concentration). A 5 nm thick alumina layer was deposited with ALD to protect the YVO4 substrate during subsequent dry etching steps. Next, a 150 nm thick film of niobium was sputtered on the surface. The sputterer has a base pressure of 5$\times {10}^{-10}$ Torr. The deposition was done at a pressure of 7 mTorr with a DC bias power of 200 W resulting in a deposition rate of ~7 Å/s. The deposition pressure was chosen to minimize the niobium thin film stress. MaN-2403 was patterned on the niobium surface using electron beam lithography to define the niobium resonator, coupling waveguide and ground plane. A 25 nm thick aluminum hard mask was deposited on the surface using electron-beam evaporation and the MaN-2403 pattern was transferred to the aluminum hard mask layer with liftoff in Remover PG. The niobium layer was etched in SF_6_ + Ar chemistry with an ICP-RIE process using aluminum pattern as the hard mask.

A 300 nm thick film of amorphous silicon was deposited by PECVD. The amorphous silicon photonic structure was defined with electron beam lithography in HSQ (FOx-16) resist. The pattern was transferred to the amorphous silicon layer using a pseudo-bosch process (SF_6_ + C_4_F_8_ chemistry) in an ICP-RIE system. An HF dip was used to remove the remaining HSQ mask and the aluminum hard mask that protected the niobium layer during the amorphous silicon etching step.

The fabricated device was mounted in a copper box with optical access through a hole in the lid of the box. The niobium co-planar waveguide and ground plane were wire-bonded to a PCB launch board that connected to coaxial cable with an SMP connector. The box was mounted on a copper post on the mixing chamber stage of the dilution refrigerator.

### Measurement setup

A detailed diagram of the measurement set-up is shown in Fig. S7. We coupled to the device optically using an optical fiber that focused light through a lens pair onto the grating coupler that coupled to the optical resonator. The optical fiber and lens pair setup were mounted on a 3-axis piezo stack to control the position of the light and aligned with a 5^o^ angle (relative the axis of the sample surface) to best match the output mode of the grating coupler.

To generate a bias magnetic field for the transducer, we used a two-axis home-built split-pair superconducting electromagnet mounted on the mixing chamber stage of the dilution refrigerator. One axis was used to generate a large (up to ~100 mT) in-plane magnetic field and the second smaller correction coil was used to minimize the out of plane magnetic field.

Gas condensation was used to tune the optical cavity into resonance with the Er^3+^:YVO_4_ optical transitions. Nitrogen gas was introduced to the fridge at 4 K (i.e. before cooling to base temperature using the dilution unit) and frozen within a copper tube. A heater, thermally lagged to the copper tube at the 4 K stage, permitted the frozen nitrogen to sublimate and then condense on the optical cavity, which provided the resonance tuning. The heater power and duration were controlled to achieve the target frequency.

For microwave measurements, a network analyzer or microwave signal generator was used as the input signal. For pulsed measurements, the microwave input passed through a fast microwave switch before the fridge. The input microwave coaxial cable to the device in the fridge passed through a series of attenuators at the different stages of the fridge (40 dB in total) before connecting to the PCB launch board with an SMP connector. The output microwave signal passed through two circulators on the mixing chamber stage, superconducting coax between the mixing chamber stage and the 4 K stage and a HEMT on the 4 K stage before exiting the fridge. A low noise amplifier (LNA) at room temperature was used to further amplify the signal before detection on a network analyzer, spectrum analyzer or digitizer when measuring weak signals.

For the optical measurements, an external cavity diode laser (ECDL) was locked to a stable reference cavity for measurements where a precise laser frequency was needed. For measurements that swept the laser frequency several GHz, the laser was left unlocked, and an internal piezo actuator was used.

The input light path was modified for the different experiments. For heterodyne measurements, the input light was split on a 90/10 beam splitter, where the 10% path acted as a LO for heterodyne detection and the 90% path passed through a 100 MHz fiber AOM, a polarization controller and a variable optical attenuator towards the device. The AOM acted as a fast optical switch for pulsed measurements and offset the transduction pump laser frequency (and thus the upconverted transduction signal) for heterodyne detection. For photon detection with an SNSPD, an additional Fabry-Perot cavity (finesse = 1000, FSR = 100 GHz) was used at the input to filter the laser noise and was locked to the laser frequency with piezo feedback. A fiber circulator was used to input light to the fridge and route the output photons from the device to the optical detection path.

For heterodyne measurements, the output optical signal was combined with the LO and mixed down to microwave frequencies on a photodiode. The photodiode output signal passed through a bias tee and the high frequency component was amplified using two LNAs before detection on a network analyzer.

For SNSPD detection, the output optical light passed through a filtering setup consisting of two high finesse fiber-coupled Fabry-Perot filter cavities (finesse = 10,000, FSR = 20 GHz), a broadband bandpass filter array and two fiber circulators before each Fabry-Perot cavity and a series of optical MEMS switches which were used to change the light path between the locking path and a detection path. The Fabry-Perot filters were frequency stabilized to the transduction light frequency using Pound-Drever-Hall locking with feedback on the Fabry-Perot piezo in a pulsed mode. Every 5 s, the light path switched from measuring transduced photons with the filter piezo voltage held at a fixed value to a locking path where light at the transduction frequency was generated from the laser with an EOM sideband and detected on a photodiode for feedback.

Before each high finesse filter, fiber circulators were used to prevent reflections and mode coupling between the filters. A broadband bandpass filter array (1× FWHM = 30 GHz filter, 3× FWHM = 400 GHz filters) was place between the two Fabry-Perot filters to prevent far detuned laser noise that leaked through our initial laser noise filter from reaching the SNSPD. Before entering the fridge, the light passed through a 2 km fiber to delay the transduction signal by 11 µs. We observed crosstalk between the optical pump to our device and the SNSPD in the same fridge, so the time delay allowed us to filter out the crosstalk in the time domain. Better packaging in future experiments can eliminate the crosstalk. The light went back into the dilution refrigerator and passed through a coiled fiber on the 4 K stage to filter IR photons before detection on the SNSPD (background counts ~5 counts/s).

The filter setup insertion loss was −15 dB, where most of loss came from the insertion loss of the Fabry-Perot filters (~ −7.75 dB). The remaining loss came from the optical MEMS switches (<−0.5 dB each), fiber circulators (<−0.5 dB each), the broadband filters (−2.2 dB), fiber mating connections, and SNSPD detection efficiency (−3 dB). The detection noise floor was 10 counts/s, which included 5 counts/s from laser leakage when ${P}_{o}$ = 550 µW. This corresponds to a filter extinction of ~ 140 dB. Depending on the pulse sequence duty cycle, we also observed PL noise leakage (see Supplementary Note 16). However, for low duty cycle measurements (i.e. off time > 10 ms), this was not a dominant factor.

For detection of the microwave resonator noise under pulsed optical excitation, the noise was mixed down to ~10–20 MHz and detected on a digitizer after exiting the fridge. For continuous wave optical light excitation, a spectrum analyzer was used for detection.

## Supplementary information

Supplementary Information

## Data Availability

The data that support the findings of this study are available from the corresponding author upon request.
