# Supplementary material for: Microwave-to-optical transduction with erbium ions coupled to planar photonic and superconducting resonators
Source: Nat Commun. 2023 Mar 1;14:1153. doi: 10.1038/s41467-023-36799-0 (PMC9977906; doi:10.1038/s41467-023-36799-0)
Supplement: Supplementary file 1 — Supplementary Information [file 41467_2023_36799_MOESM1_ESM.pdf]

Supplementary Information:

Microwave-to-optical transduction with erbium ions coupled to planar photonic and superconducting resonators

Rochman et al.

Supplementary Note 1. Rare-earth ion cavity coupling theory

The coupling between a cavity and ensemble of ions was simulated for both microwave and optical frequencies using the model described in Ref [1]. A cavity mode, $\hat{a}$, with a resonance frequency of $\omega_{c,0}$ is coupled to N two-level atoms $\sigma_z(\omega_k)$ in the weak excitation regime. The Hamiltonian is:

$$H = \hbar\omega_0 \hat{a}^\dagger \hat{a} + \sum\nolimits_k \frac{1}{2}\hbar\omega_k \sigma_{z,k} + i\hbar \sum_k^N g_k (\hat{a}^\dagger \sigma_{-,k} - \sigma_{+,k}\hat{a}) \quad (1)$$

Here, g_k describes the coupling strength between $\hat{a}^\dagger$ and $\sigma_{-,k}$. With input-output theory, the transmission of the input field is:

$$t(\omega) = \frac{\kappa/2i}{\omega - \omega_0 + \frac{i\kappa}{2} - W(\omega)}, \quad (2)$$

where the cavity-ensemble coupling term is given by $W(\omega) = \frac{\sum_k g_k^2 (n_{1,k} - n_{2,k})}{\omega - \omega_{\text{ion}} + \frac{i\gamma}{2} + i\Delta}$ for an ensemble with a Lorentzian inhomogeneity line shape that is centered at ω_{ion} with a FWHM, Δ, and a mean population within the two energy levels of $n_{1,k}$ and $n_{2,k}$. The cavity and atomic decay rates are denoted by κ and γ, respectively. The poles of the transmission spectrum can be found at:

$$\omega_\pm = \frac{\omega_{c,0} + \omega_{\text{ion}}}{2} - \frac{i\gamma + i\kappa + 2i\Delta}{4} \pm \sqrt{\sum\nolimits_k g_k^2 (n_{1,k} - n_{2,k}) + \left(\frac{2(\omega_{ion} - \omega_0) - i\gamma - 2i\Delta + i\kappa}{4}\right)^2} \quad (3)$$

The real component of the pole represents the frequency of the coupled modes. In the ensemble strong coupling regime, the coupled modes are split into two polariton modes. The imaginary component represents the linewidth the modes.

Supplementary Note 2. REI transduction theory

The system we have for the transducer can be approximated as an ensemble of three level atoms coupled to three electromagnetic fields (see Figure S1). Two of these fields correspond to an optical and microwave cavity mode, which are the input and output modes of the transducer, while the third field corresponds to an optical laser field that compensates for the frequency difference between the input and output fields.

In order to model this atom-cavity system, we can use the following Hamiltonian [2]:

$$\frac{H}{\hbar} = \delta_{c,o}\hat{a}^\dagger\hat{a} + \delta_{c,\mu}\hat{b}^\dagger\hat{b} + \sum_{k}^{N} \delta_{\mu,k}\sigma_{22,k} + \delta_{o,k}\sigma_{33,k} + (g_{\mu,k}\hat{b}\sigma_{21,k} + \Omega_{\mathrm{o,k}}\sigma_{32,\mathrm{k}} + g_{o,k}\hat{a}\sigma_{31,k} + \mathrm{H.C.}) \tag{4}$$

where $\delta_{c,o}$ is the optical light-cavity detuning, $\delta_{c,\mu}$ is the microwave light-cavity detuning, $\delta_{\mu,k}$ is the detuning between the kth atom and the microwave field, $\delta_{o,k}$ is the detuning between the kth atom and the optical field, $g_{\mu,k}$ is the coupling strength between the kth atom and the microwave cavity, $\Omega_{\mathrm{o,k}}$ is the optical pump Rabi frequency of the kth atom, $g_{o,k}$ is the coupling strength between the kth atom and the optical cavity, H. C. is the Hermitian conjugate and N is the number of atoms. We denote the optical cavity by the annihilation operator, $\hat{a}$, the microwave cavity by the annihilation operator, $\hat{b}$, and $\sigma_{ij} = |i\rangle\langle j|$ are the operators of the atoms. This Hamiltonian is suited for ground state transduction, but it can be reformulated in a similar fashion for excited state transduction.

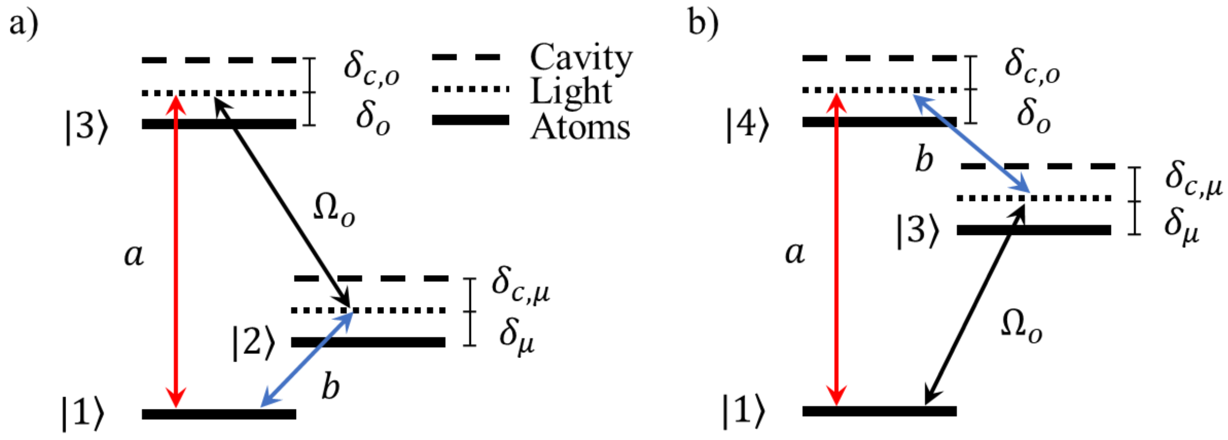

Figure S1: Energy level diagram of a REI transducer using the a) ground spin state and b) excited spin state.

We are interested in determining the transduction efficiency for this system. That is $\eta = |A_{\mathrm{out}}/B_{\mathrm{in}}|^2$ or $\eta = |B_{\mathrm{out}}/A_{\mathrm{in}}|^2$, where A_{in} and B_{in} are the cavity input fields and A_{out} and B_{out} are the cavity output fields for cavity modes $\hat{a}$ & $\hat{b}$, respectively. We can start with the equations of motion of the two cavity fields in terms of this Hamiltonian:

$$\dot{\hat{a}} = -i[\hat{a}, H] - \frac{\kappa_o}{2} - \sqrt{\kappa_{o,\mathrm{in}}} A_{\mathrm{in}} \tag{5}$$

$$\dot{\hat{b}} = -i[\hat{b}, H] - \frac{\kappa_\mu}{2} - \sqrt{\kappa_{\mu,\mathrm{in}}} B_{\mathrm{in}} \tag{6}$$

where κ_μ and κ_o are the total microwave and optical cavity decay rates, respectively, and $\kappa_{\mu,\mathrm{in}}$ and $\kappa_{o,\mathrm{in}}$ are the microwave and optical input cavity decay rates, respectively. In general, solving these equations exactly is challenging due to the large number of atoms, so we used an approximated form to make the calculation easier.

Adiabatic model

In order to gain some intuition for the transducer efficiency, we can make an adiabatic approximation to find a relatively simple analytical solution for the transducer Hamiltonian in the steady state [2]. The transduction efficiency can be determined to be:

$$\eta = \left| \frac{2R}{R^2 + 1} \right|^2 \tag{7}$$

where $R = 2S/\kappa_o \kappa_\mu$. S is the effective linear coupling strength between the microwave and optical cavity in the adiabatic limit, which can be expressed as:

$$S = \sum_k^N \frac{\Omega_k g_{\mu,k} g_{o,k}^*}{\delta_{o,k} \delta_{\mu,k}} = \frac{\alpha F \Omega_{\mathrm{max}} \sqrt{\omega_o \omega_\mu}}{2} \tag{8}$$

where α contains the spectroscopic parameters of the ions, F is the mode overlap between the three fields and Ω_{max} is the maximum optical Rabi frequency of the pump field. We note that $S \propto \frac{N d_{31} d_{32} \mu_{21}}{\Delta_o \Delta_\mu}$, where d_{ij} and μ_{ij} are the dipole moments of the atomic transition between level *i* and j, *N* is the number of atoms and Δ_o and Δ_μ are the optical and microwave transition inhomogeneous linewidths. In the adiabatic limit, the detunings will be proportional to the inhomogeneous linewidths.

Linear model

We can solve the transducer Hamiltonian in a more general case (i.e. not limited to the adiabatic limit) using a master equation-based model and a linear approximation for the cavity fields [3]. This model allows us to incorporate atomic energy and dephasing loss mechanisms and solve the Hamiltonian for choice of detunings. The linear approximation is appropriate for small cavity fields and makes the system computationally easier to solve.

The dynamics of the system can be described by:

$$\frac{d}{dt}\rho = -i[H, \rho] + L \tag{9}$$

where ρ is the density matrix of each atom and L describes energy loss and dephasing of the atoms. L can be written as the sum of the different contributions by $L = \sum_i L_{ij}$, where L_{ij} describes the energy loss or dephasing between energy levels *i* and *j*. We can write the density matrix as a 9x1 vector, $\boldsymbol{\rho}$, and the Hamiltonian interactions and Lindblad decay processes as a 9x9 matrix, $\boldsymbol{L}$, such that $\dot{\boldsymbol{\rho}} = \boldsymbol{L\rho}$ [4].

We can make a linear approximation to make this system of equations easier to compute [3]. First, L can be rewritten without approximation in terms of its linear dependence on the two cavity fields:

$$\boldsymbol{L} = \boldsymbol{L_0} + a\boldsymbol{L_a} + a^*\boldsymbol{L_{a^*}} + b\boldsymbol{L_b} + b^*\boldsymbol{L_{b^*}} \tag{10}$$

Next, we make an approximation that our density matrix for each atom depends linearly on the cavity field amplitudes, which should be true for sufficiently small cavity field amplitudes:

$$\boldsymbol{\rho} = \boldsymbol{\rho_0} + a\boldsymbol{\rho_a} + a^*\boldsymbol{\rho_{a^*}} + b\boldsymbol{\rho_b} + b^*\boldsymbol{\rho_{b^*}} \tag{11}$$

From here, we can solve for $\boldsymbol{\rho}$ in the steady state using $L_0\boldsymbol{\rho_0} = 0$, $\mathrm{Tr}(\boldsymbol{\rho}) = 1$ and $\boldsymbol{\rho_x} = -\boldsymbol{L_0^{-1}L_x\rho_0}$ for $x = a, b, a^*, b^*$. We can use this result to find the cavity field amplitudes. Importantly, these equations result in a linear coupling between the two cavity modes and can be expressed in matrix form:

$$\begin{bmatrix} -\sqrt{\kappa_{o,\mathrm{in}}}A_{\mathrm{in}} \\ -\sqrt{\kappa_{\mu,\mathrm{in}}}B_{\mathrm{in}} \end{bmatrix} = \begin{bmatrix} i\delta_{c,o} + \kappa_o + iS_{a,31} & iS_{b,31} \\ iS_{a,21} & i\delta_{c,\mu} + \kappa_\mu + iS_{b,21} \end{bmatrix} \begin{bmatrix} a \\ b \end{bmatrix} \tag{12}$$

where $S_{a,ij} = \sum g_{\mu,k}\rho_{a,ij,k}$ (and similar for $S_{b,ij}$). We can solve this system of equations analytically and find the transduction efficiency equation:

$$\eta_d = \left|\frac{A_{\mathrm{out}}}{B_{\mathrm{in}}}\right|^2 = \left|\frac{iS_{b,31}\sqrt{\kappa_{o,\mathrm{in}}\kappa_{\mu,\mathrm{in}}}}{S_{a,21}S_{b,31} + (i\delta_{c,o} + \kappa_o + iS_{a,31})(i\delta_{c,\mu} + \kappa_\mu + iS_{b,21})}\right|^2 \tag{13}$$

Supplementary Note 3. Resonator design and simulations

The geometric parameters of the optical resonator are summarized in Table S1 and the mirror geometry is shown in Figure S2. The optical cavity consists of a 100 µm long waveguide between two photonic crystal mirrors that are patterned out of amorphous silicon on the Er^{3+}:YVO_4 surface. The amorphous silicon waveguide has a height of 300 nm and a width of 600 nm. One of the optical mirrors has a shorter length (i.e. 2 mirror periods) that we used for coupling, while the second mirror has a longer length (i.e. 25 mirror periods) that we used for high reflectivity. The photonic crystal mirror consists of ellipses with a pitch, a_o, and radii of $r_{\parallel,0}$ and $r_{\perp,0}$ .The photonic crystal mirrors mode is tapered to the waveguide mode by reducing the size of the photonic crystal mirror holes linearly over 15 periods on both sides of the two mirrors to a final period of, a_t, and radii of $r_{\parallel,t}$ and $r_{\perp,t}$, as shown in Figure S2. Coupling to the resonator from free space was done with a grating coupler and optical waveguide. Two grating couplers are patterned with the resonator, but for experiments we only couple to the grating connected to the low reflectivity mirror and measure the reflected signal. The magnetic field of the optical waveguide mode, as shown in Figure S3, is the relevant field for our experiments because we use the magnetic dipole moment for the optical transitions in the transducer.

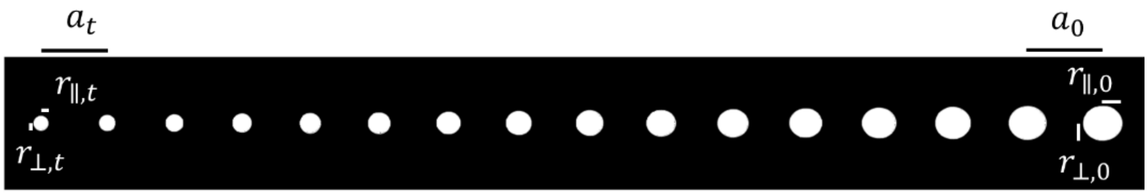

Figure S2: Photonic crystal geometry. The geometric parameters that define the photonic crystal mirror including the mirror period and radii and the final taper period and radii.

Table S1: Optical resonator geometric parameters

Optical Resonator Parameter	Value
Waveguide height	300 nm
Waveguide width	600 nm
Waveguide length	100 μm
a_o	350 nm
$r_{\|,0}$	95 nm
$r_{\perp,0}$	105 nm
a_t	315 nm
$r_{\|,t}$	20 nm
$r_{\perp,t}$	20 nm
Mirror periods	2,25
Taper periods	15

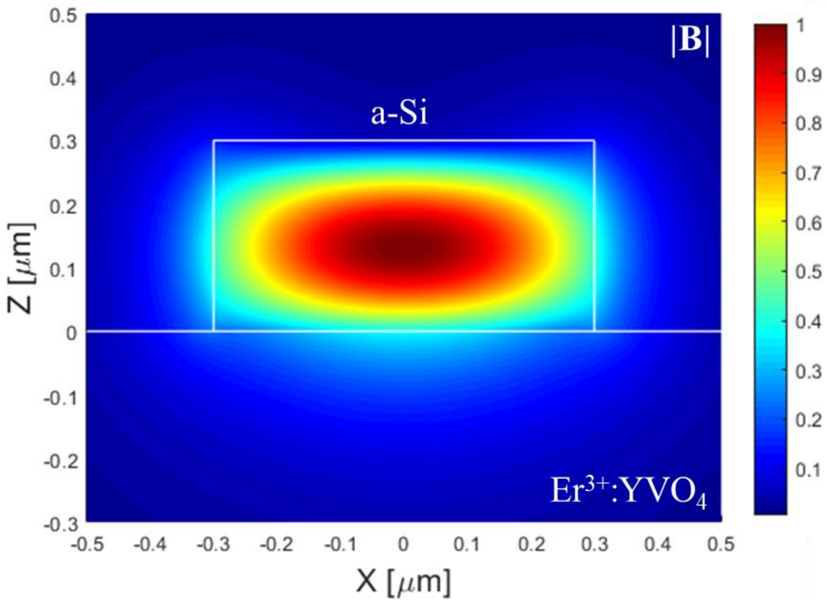

Figure S3: Normalized magnetic field profile of the amorphous silicon waveguide TM mode. The dominant component is B_x, which is what we use to couple to the erbium optical transitions.

The geometric parameters of the microwave resonator are included in Table S2 and the mode profile is shown in Figure S4. The microwave resonator is made from 150 nm thick film of niobium that is patterned on the surface. It consists of a 100 µm long, 1 µm wide inductive wire, that we used to confine the microwave magnetic field to the optical resonator, and an interdigitated capacitor to set the resonance frequency of the cavity. The geometry is similar to other low impedance microwave resonators used for coupling to spins [5,6]. Coupling to the microwave cavity was done with a co-planar microwave waveguide that is 4 µm away from the interdigitated capacitor. The optical cavity was patterned 1.2 µm away from the microwave cavity when measured edge-to-edge.

Table S2: Microwave resonator geometric parameters

Microwave Resonator Parameter	Value
Inductive wire length, L_{ind}	100 µm
Inductive wire width, W_{ind}	1 µm
Capacitor finger length, L_{cap}	485 µm
Capacitor finger width, W_{cap}	10 µm
Capacitor finger gap, G_{cap}	5 µm
Capacitor finger number, N_{cap}	20
Optical gap width, $G_{o,w}$	235 µm
Optical gap height, $G_{o,h}$	55 µm
Waveguide coupling gap, $G_{wg,\mathrm{cou}}$	4 µm
Waveguide width, W_{wg}	10 µm
Waveguide gap, G_{wg}	4.5 µm

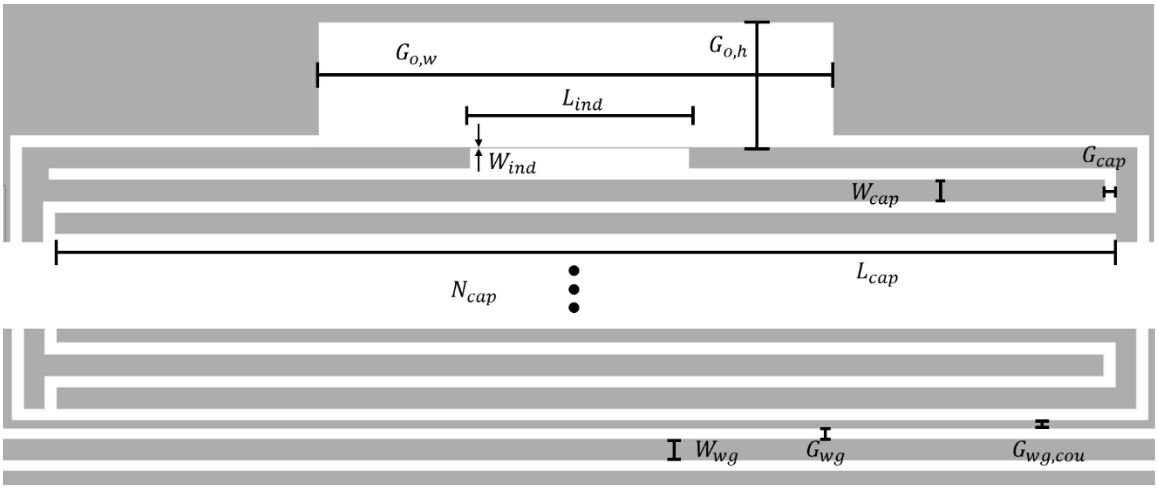

Figure S4: Microwave cavity geometry. The geometric parameters that define the pattern of the microwave resonator including the parameters related to the inductive wire, the interdigitated capacitor, the coupling waveguide and the gap for the optical resonator.

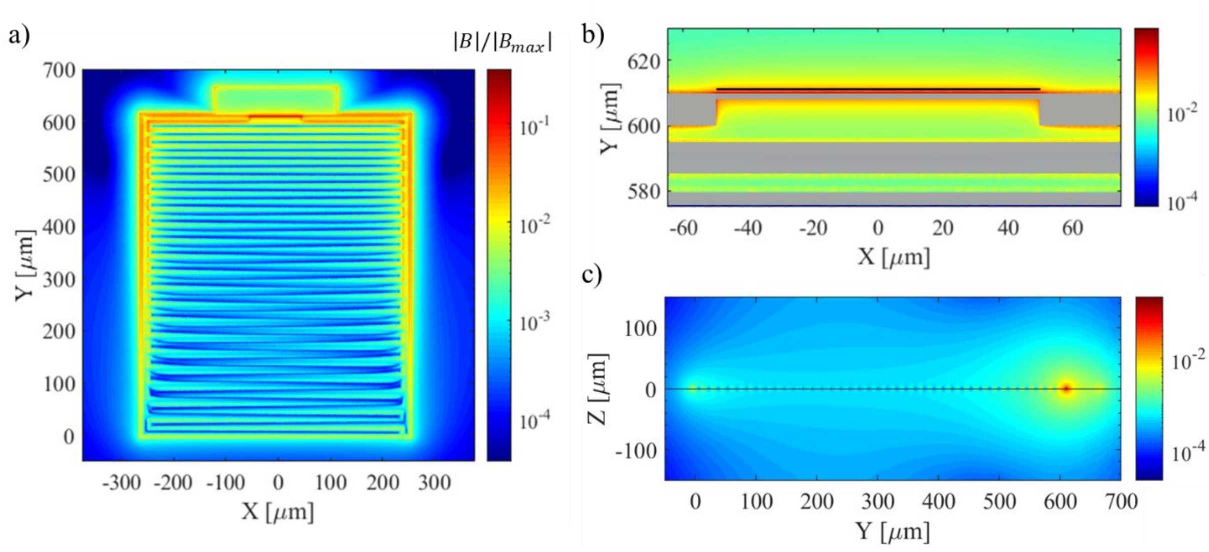

Figure S5: The normalized magnetic field distribution of the microwave resonator. a) The magnetic field distribution in the plane of the niobium film. The dominant component is B_z at the optical resonator position, which is used for coupling to the erbium spin transitions. b) A closer look at the magnetic field distribution near the inductive wire. The black line indicates the location of the optical resonator for reference. c) The cross-section of the microwave resonator magnetic field.

Supplementary Note 4. Atomic system transduction simulations

We utilize optical transitions between the Z_1 crystal field levels within the $^4I_{15/2}$ manifold and the Y_1 crystal field levels within the $^4I_{13/2}$ for the transducer. Electron spin transitions in the Z_1 doublet are used for ground state transduction and transitions in the Y_1 doublet are used for excited state transduction.

Bias magnetic field angle

As presented in Ref [7], sigma-polarized light can drive all four Z_1-Y_1 transitions when the DC bias magnetic field is parallel to the c-axis. The spin-flipping transitions are electric-dipole (ED) transitions and the spin-preserving transitions are magnetic-dipole (MD) transitions. In this magnetic field configuration, transduction cannot occur when coupling the ions to a standing-wave cavity. This is because the cavity mode overlap factor [2]:

$$F \equiv \frac{1}{\sqrt{V_\mu V_o}} \left| \iint \chi(r)\psi(r)\phi(r) d^3r \right| \tag{14}$$

V_μ, V_o: microwave, optical mode volume respectively

$\chi(r), \psi(r), \phi(r)$: microwave, optical, and optical pump modes respectively

will vanish if the two optical transitions are ED and MD, because the electric field and magnetic field inside the standing-wave cavity are 90° out of phase. Therefore, to avoid a vanishing mode overlap factor, we applied the DC magnetic field at an angle between crystal c-axis and a-axis to mix the electronic states. Therefore, both ED and MD transitions are allowed for all four transitions in Z_1-Y_1 manifold.

A simulation was performed to study the state mixing effect at different magnetic field angles, as showed in Figure S6, following the methodology described previously [8]. The figure of merit is $|d_{31}d_{32}|^2$ as the transducer efficiency scales with this factor. A magnetic field angle of 35° from the c-axis is optimal for mixing the states.

Beyond optimizing the dipole moment product, the magnetic field angle determines the g-factors of Z_1 and Y_1, which need to be sufficiently different to have isolated optical transitions. When the magnetic field angle from the c-axis is greater than 45°, the dispersive shift of the microwave cavity when the excited state spins are resonant with the microwave cavity is <10 MHz. Therefore, we set the magnetic field angle to be 50° from the c-axis in the experiment to have sufficient state mixture of the electronic states and enough difference between the g-factors of Z_1 and Y_1. The spectroscopic parameters of the Z_1-Y_1 transition at the 50° angle are shown in Table S3 based on the values from [7,9].

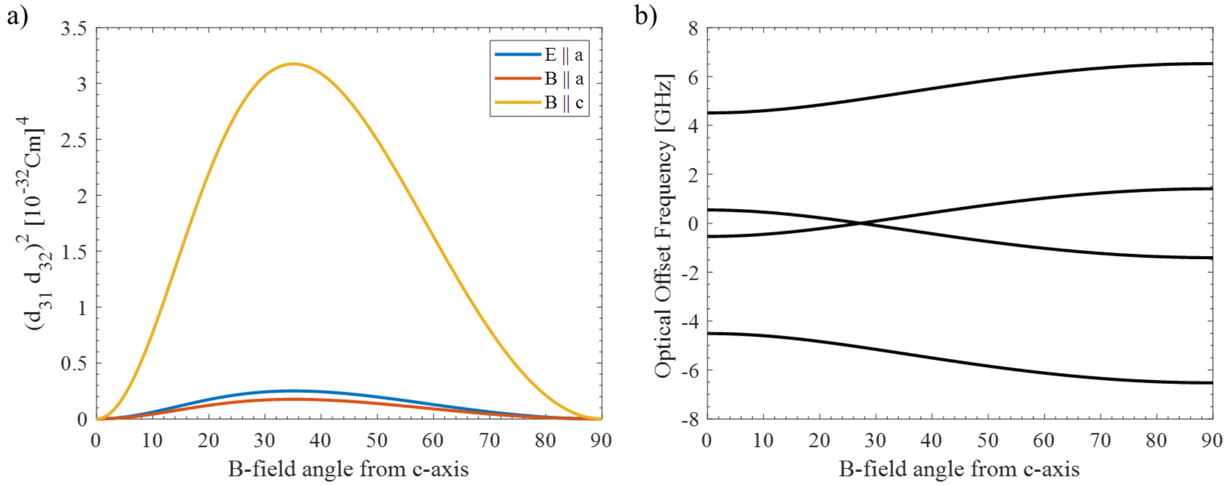

Figure S6: a) Dipole moment product for the different transition dipole operators (i.e. electric or magnetic) and orientations (i.e. parallel to a-axis or c-axis) as a function of the applied magnetic field angle. b) Optical frequencies of the four different transitions as a function of the magnetic field angle ($|B| = 80$ mT).

Table S3: Spectroscopic parameters for the Z_1-Y_1 transition at a magnetic field angle 50° from the c-axis.

Parameter	Value
g_{dc}, Z_1	5.89
g_{dc}, Y_1	4.55
$d_{B\parallel c,\perp}$ $[10^{-32}\ \mathrm{C}\cdot\mathrm{m}]$	0.48
$d_{B\parallel c,\perp}[10^{-32}\ \mathrm{C}\cdot\mathrm{m}]$	3.26
$d_{B\parallel c,\perp}[10^{-32}\ \mathrm{C}\cdot\mathrm{m}]$	1.58
$d_{B\parallel c,\perp}[10^{-32}\ \mathrm{C}\cdot\mathrm{m}]$	0.23
$d_{B\parallel c,\perp}[10^{-32}\ \mathrm{C}\cdot\mathrm{m}]$	0.52
$d_{B\parallel c,\perp}[10^{-32}\ \mathrm{C}\cdot\mathrm{m}]$	0.86

Simulated transduction efficiency

In order to simulate the transducer efficiency, we follow the master equation model with a linear approximation as outlined above. Computationally, this requires finding the inverse of N_{sim} 9x9 matrices, where N_{sim} is the number of atoms in the simulation, and some additional matrix multiplication.

When implementing this model we make a few assumptions. First, we assume the microwave coupling for the spins within the optical cavity is constant. Second, there are far too many ions to have a unique density matrix for each atom. To overcome this, we simulate $N_{sim} = 10^6$ unique ions and assume these ions are representative of the ensemble in terms of the inhomogeneity of the parameters and scale their result (i.e. density matrix) for the rest of the ions. We also ignore all the other levels within the Er^{3+}:YVO_4 level structure for simplicity and assume there are no ^{167}Er isotope ions.

The parameter inhomogeneity that is modelled includes the spectral inhomogeneities, of both the spin and optical transitions, and the inhomogeneity in the optical coupling, $g_{o,k}$, and equivalently in the optical pump Rabi frequency, $\Omega_{o,k}$. The distributions of these three parameters are randomly sampled over N_{sim} number of ions. The $g_{o,k}$ distribution is determined from the optical cavity magnetic field distribution within the Er^{3+}:YVO_4 material (see Figure S3). We assume Lorentzian distributions for the optical and microwave transition inhomogeneities.

With this methodology, the device parameters in Table S4, and the system calibration detailed in Section 6, we simulate the transducer and predict a total device efficiency of $\eta_d = 1.1 \cdot 10^{-7}$ at an optical pump power of 550 μW. This is similar to the measured value of $\eta_d = 8 \cdot 10^{-8}$. This difference can be attributed to several possible factors. First, we are not accounting for the ^{167}Er hyperfine transitions in the simulation, which could degrade the efficiency slightly, even though in the experiment we choose the magnetic field to minimize their detrimental effect. Also, the optical and spin dephasing rates used in the simulation (i.e. $\gamma_{2\mathrm{D}} = \gamma_{3\mathrm{D}} = 2\pi \cdot 10\ \mathrm{MHz}$) and the excited state spin linewidth are assumed values to best match the experimental data. Further spectroscopy is needed to verify their values in the precise experimental configuration used here.

In order to compare our transducer to other rare-earth ion transducers, we can also calculate the theoretical effective linear coupling strength, S, of our transducer. Following the notation in Williamson et al [2], from our spectroscopy, we obtain $\alpha = 1.54 \cdot 10^{-10}$ s for detunings of three inhomogeneous linewidths. From electromagnetic simulation of the cavities, we obtain a mode overlap $F = 9.5 \cdot 10^{-4}$

and a maximum optical pump Rabi frequency of $\Omega_{\max} = 2\pi \cdot 11.5$ MHz (for an intracavity photon number of $\langle n_o \rangle \sim 10^4$), which results in $S = 2\pi \cdot 6$ MHz.

Table S4: Parameters of device for simulation

**Parameter**	**Value**
Optical lifetime	3.3 ms[*]
Optical dephasing rate, $\gamma_{3\mathrm{D}}/2\pi$	10 MHz[X]
Optical inhomogeneous linewidth, Δ_o	300 MHz[*]
Spin lifetime	1 ms[*]
Spin dephasing rate, $\gamma_{2\mathrm{D}}/2\pi$	10 MHz[X]
Spin inhomogeneous linewidth, Δ_o	65 MHz[*]
Optical cavity input decay rate, $\kappa_{o,\mathrm{in}}/2\pi$	2.9 GHz[!]
Optical cavity total decay rate, $\kappa_{o,\mathrm{tot}}/2\pi$	13.2 GHz[!]
Microwave cavity input decay rate, $\kappa_{\mu,\mathrm{in}}/2\pi$	0.85 MHz[!]
Microwave cavity total decay rate, $\kappa_{\mu,\mathrm{tot}}/2\pi$	2 MHz[!]
Maximum optical pump Rabi frequency, $\Omega_{o,\max}/2\pi$	11.5 MHz[+]
Maximum optical ion cavity coupling rate, $g_{o,\max}/2\pi$	783 kHz[+]
Spin cavity coupling (for transducer ions), $g_{\mu,1}/2\pi$	255 Hz (165 Hz)[+]
Total spin coupling, $g_{\mu,\mathrm{tot}}/2\pi$	120 MHz (91 MHz)[+]
Ions in optical cavity	2.1e8[+]
Ions in microwave cavity	1.6e15[+]
Temperature	100 mK[X]
Optical ion-cavity detuning (zero field)	1.8 GHz[!]

Values in the brackets correspond to excited state parameters, while the nominal value is for the ground state. The parameters were determined from spectroscopy ([*]), assumed value ([X]), measurement ([!]) or simulation ([+]).

Supplementary Note 5. Set up diagram

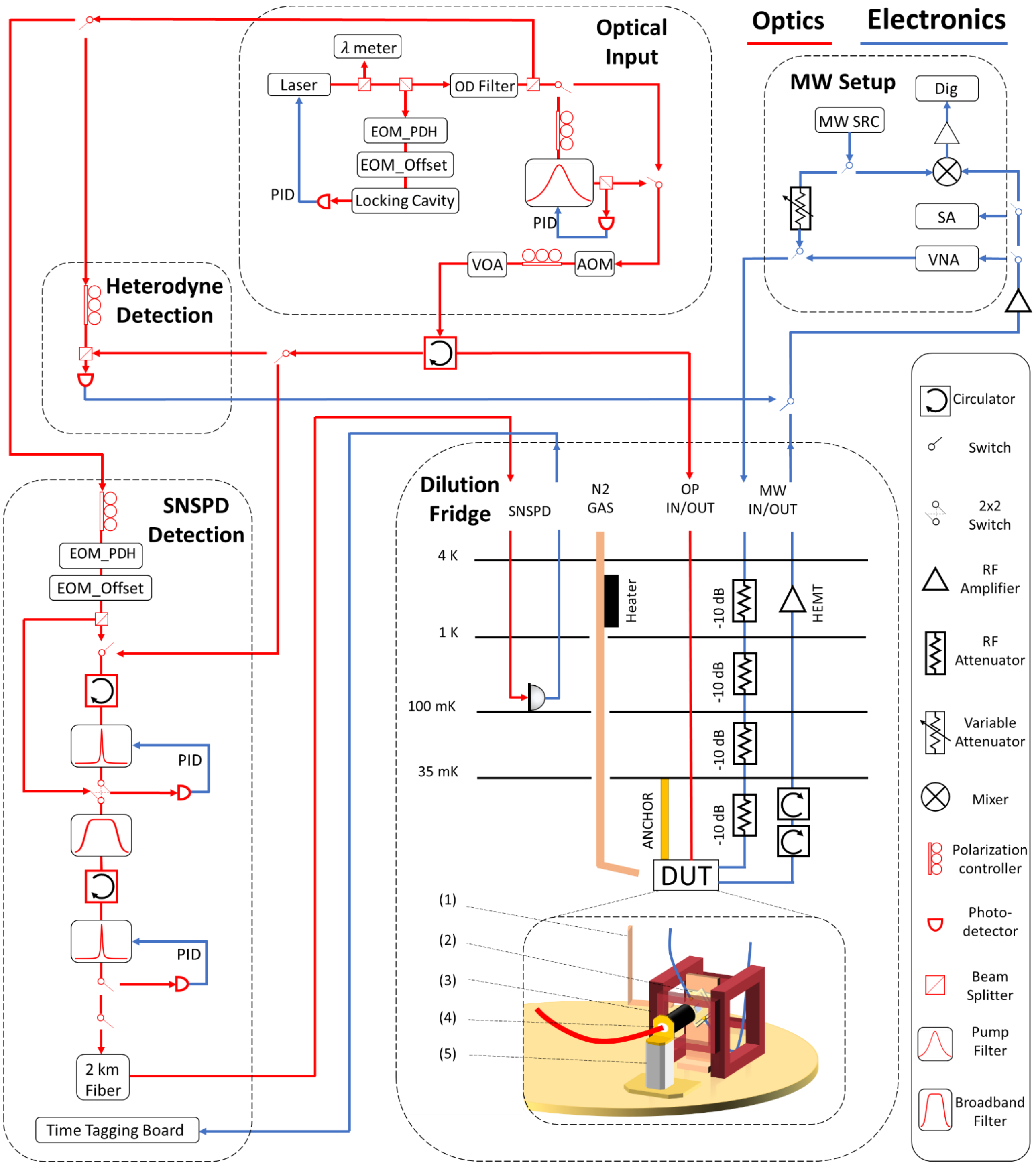

Figure S7: Diagram of the full experimental set-up. This includes the optical and microwave signal generation, the dilution fridge setup, and the optical and microwave signal detection. The device setup in the fridge consists of: 1) gas tuning line, 2) microwave coax, 3) superconducting magnet, 4) optical fiber and lens tube, and 5) three-axis piezo stack. Further details are in the text.

Supplementary Note 6. Efficiency calculations

Efficiency calibrations

The detected device efficiency is determined by the ratio of the output optical photons in the optical fiber compared to the input photons in the superconducting microwave coupling waveguide.

For the microwave input, we needed to calibrate the microwave losses of the input microwave signal from the microwave source to the superconducting coupling waveguide. We calibrated the input microwave loss to be 46 dB which includes 40 dB loss from 4x 10 dB attenuators and 6 dB of cable loss.

We account for the losses of the 50/50 fiber beam splitter and fibers that we used to combine the transduced signal and the local oscillator (which is the -3 dB loss of the 50/50 beam splitter and -1.5 dB of excess loss). We also account for electrical losses of the bias tee and coaxial cable from the photodetector to the amplifiers and network analyzer (-1.65 dB).

To calculate the pulsed transduction device efficiency from SNSPD detection, we compared the detected photon counts to the input photon number given the input power level referenced to the input microwave waveguide, pulse duration and the number of input pulses. We account for the filter detection losses and SNSPD detection efficiency, 15 dB in total, to determine the device efficiency. The input microwave loss is 4 dB larger compared to the previous CW heterodyne measurements because we have added a microwave switch and an additional coaxial cable. In order to determine the detected photon number, we integrate the transduction signal over the time bins associated with the transduction pulse. The noise level is negligible for most measurements, but for the lower SNR measurements, the noise level is subtracted out to prevent overestimating the efficiency.

Internal efficiency

In order to estimate the device internal efficiency from the total device efficiency, we need to take into account the optical and microwave cavity coupling strength compared to all loss channels (i.e. $\kappa_{\mathrm{in}}/\kappa$) and the grating efficiency. The optical cavity coupling ratio is estimated to be $\kappa_{o,\mathrm{in}}/\kappa_o$ = 0.22 from a phase-sensitive measurement of the reflection spectrum. The optical grating coupler efficiency was measured to be 30% by measuring the reflected light from the device. The microwave cavity coupling ratio depends on the measurement regime as the microwave internal quality factor varies (see Section 8) but ranges between $\kappa_{\mu,\mathrm{in}}/\kappa_\mu$ = 0.25-0.4, where $\kappa_{\mu,\mathrm{in}}/\kappa_\mu$is closer to 0.25 in the colder device temperature experiments and is closer to 0.4 for the higher device temperature experiments. This makes the difference between the internal device efficiency and the total device efficiency to be $\eta_d/\eta_{d,\mathrm{int}}$~1.7%-2.6% (i.e. we estimate the internal efficiency is a factor of ~50x larger than the device efficiency). Efficiencies presented throughout are the total device efficiency.

Supplementary Note 7. Internal microwave quality factor vs transduction parameters

Before the transduction process, we condensed nitrogen gas on the device during the process of tuning the optical cavity in resonance with the Z_1-Y_1 transitions. During the transduction process, we applied an in-plane magnetic field of ~60-80 mT and optical power incident on the optical device. We quantified how these different factors affected the internal quality factor of the microwave resonator.

The internal microwave quality factor as a function of the circulating photon number in the microwave cavity is shown in Figure S8 for four distinct configurations. The quality factor at the initial cool down to the base temperature is shown in the blue trace. After gas tuning the optical resonator (red trace), we see a decrease in the low microwave power internal quality factor from $Q_{\mu,i}$~50,000 to ~30,000, while the quality factor at high microwave power remains largely unchanged. We also observe a shift in the resonance frequency of ~1.5 MHz due to the permittivity of condensed nitrogen being slightly larger than vacuum.

After adding the 78 mT magnetic field (yellow trace), the internal quality factor drops significantly at low microwave power. We attribute this to the ^{167}Er isotope ions that have hyperfine transitions that overlap with the microwave cavity frequency around this magnetic field (see Figure S10). The absorption of the spins saturates at high microwave power and we can recover a high internal quality factor.

Lastly, we added 1 mW of optical power resonant with the optical cavity at the same magnetic field (purple trace). At low microwave power, we observe an increase in the internal quality factor compared to without applying any optical power. We attribute this to the optical power heating up the chip, which saturates the hyperfine transition absorption that is limiting the quality factor. At high microwave power, the internal quality factor is lower compared to the case of no optical power. We attribute this to the fact that the high microwave power is used to saturate the absorption, so now the quasi-particle related losses from the optical power dominate and reduce the quality factor.

Next, we measured the microwave internal quality factor a function of the optical power at three different microwave power levels as shown in Figure S8. This was conducted after gas-tuning and with a magnetic field at 78 mT. At low microwave power (corresponding to $\langle n_\mu \rangle \sim 10^4$), increasing the optical power up to P_o~500 µW increases the internal microwave quality factor, which we attribute to a steady increase in the device temperature. The optical power increases the internal quality factor from $Q_{\mu,i}$ = 2,800 at no optical power to a maximum value of $Q_{\mu,i}$ = 22,000. Above P_o= 500 µW, the quasi-particle losses dominate, and the quality factor decreases again.

At high microwave power ($\langle n_\mu \rangle \sim 10^{10}$), increasing the optical power does not increase $Q_{\mu,i}$ as the high microwave power is already saturating the absorption. We just observe the quasiparticle related losses once they start to dominate above $P_o \sim$30 µW and see the internal quality factor decrease.

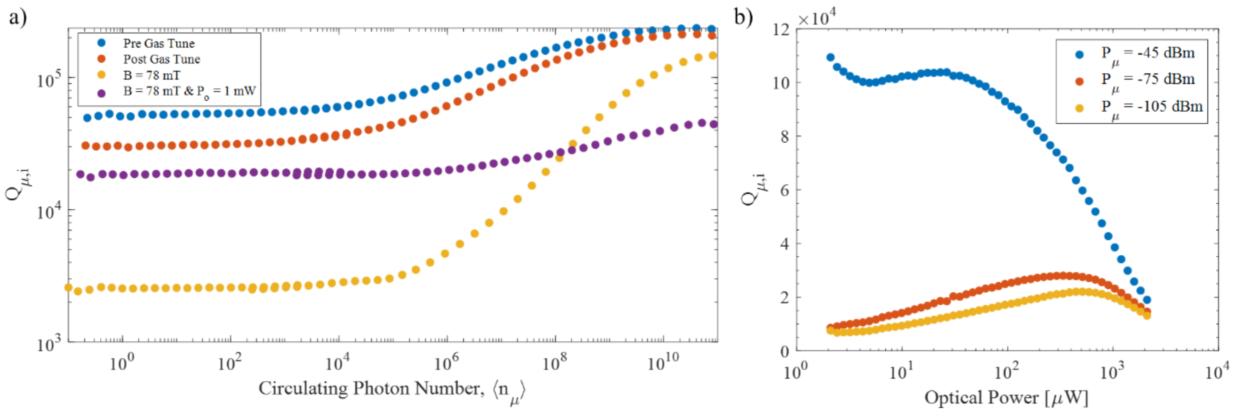

Figure S8: Microwave quality factor. a) Microwave internal quality factor as a function of the circulating photon number in four distinct regimes. These include: before gas tuning the optical device (blue trace), after gas tuning the device (red trace), in a magnetic field of 78 mT (yellow trace) and in a 78 mT magnetic field and 1 mW of optical power applied to the optical device (purple trace). b) The microwave internal quality factor as a function of the input optical power to the optical device. The different traces correspond to different microwave powers applied to the microwave resonator.

Supplementary Note 8. Optical photoluminescence spectrum & M2O laser frequency dependence

We measured the optical transition frequency inhomogeneity of the erbium ions that interact with the optical cavity mode (i.e. ions near the surface of the YVO_4 substrate) using photoluminescence. As showed in Figure S9 (blue trace), the optical inhomogeneity is around 300 MHz. This is slightly larger than the measured 260 MHz linewidth in the bulk transmission spectroscopy for the same erbium concentration.

We also determined the excited state transduction efficiency as a function of the laser frequency, as shown by the red trace in Figure S9. The optimal laser frequency for transduction is detuned from the atomic transition by ~100 MHz, which is less than the optical transition inhomogeneity.

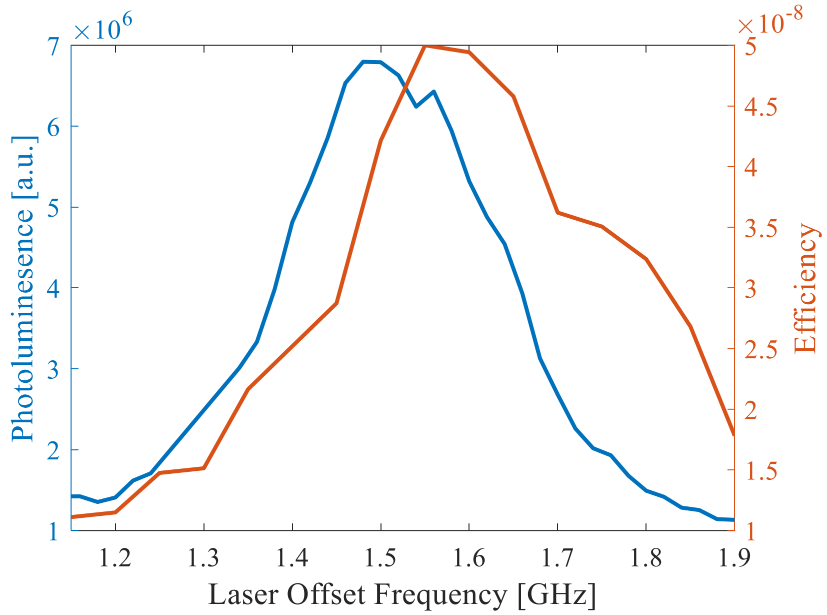

Figure S9: Transduction efficiency and optical PL as a function of the laser frequency.

Supplementary Note 9. Couplings between ^{167}Er and microwave resonator

Due to the 23% natural abundance of ^{167}Er (with nuclear spin I=7/2), we have ~120 ppm ^{167}Er within our 560 ppm crystal, which couple to the microwave resonator as shown in Figure S10. To understand the coupling structure, we use the hyperfine parameters reported in [9] to simulate the Er^{3+}:YVO_4 ground state hyperfine levels with a magnetic field angle of 50° from the c-axis. We include $\sigma_x \otimes I_N$ and $\sigma_z \otimes I_N$ operators to account for the selection rules [7,10]. The simulation and data are shown in Figure S10, where we plot the expected hyperfine transition as the red dashed lines. The simulated hyperfine transitions match well with the non-zero spin coupling structure we observe with the microwave cavity.

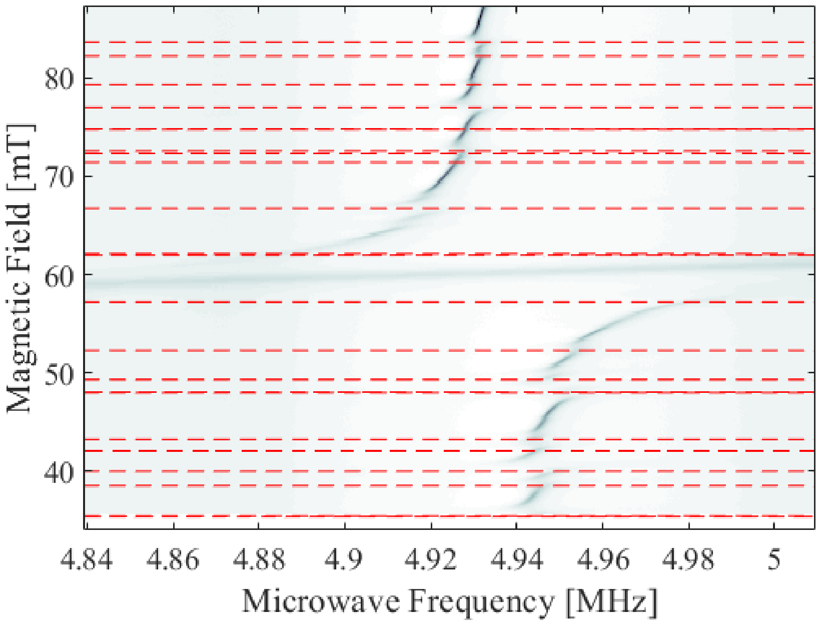

Figure S10: Microwave spin coupling with overlay of ^{167}Er hyperfine transitions.

Supplementary Note 10. Excited state pulsed M2O magnetic field dependence

The even isotope excited state spin transition that we use for transduction is at a similar microwave frequency as one of the ^{167}Er hyperfine transitions at the magnetic field used for excited state transduction. This allows the ^{167}Er ions to act as parasitic ions that can absorb microwave photons, but not transduce them, at magnetic fields where the hyperfine transitions are resonant with the microwave cavity.

Experimentally, the hyperfine transitions are avoided by detuning the magnetic field strength to the value that optimized the transduction efficiency. Under the nominal experimental condition (i.e. P_o =550 µW, P_μ =-60 dBm, $\tau_{\text{pulse}} = 1$ µs and τ_{off} =10 ms), a magnetic field sweep versus excited state transduction efficiency was measured as shown in Figure S11. The microwave frequency is optimized for each magnetic field value. The on-resonance coupling between the excited state spin and the microwave resonator is expected at 78 mT. Qualitatively, the signal shape is composed of a broad peak corresponding to the excited state coupling to the microwave cavity, with dips in efficiency corresponding to magnetic fields where hyperfine transition couple to the microwave cavity.

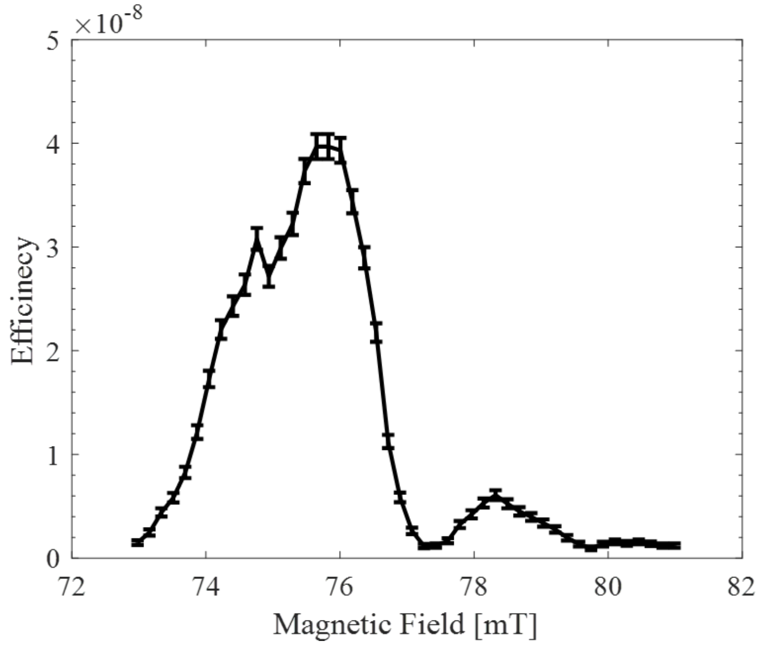

Figure S11: Excited state transduction efficiency as a function of the magnetic field strength. Error bars correspond to $\sqrt{\text{counts}}$ measured on the SNSPD and the error bars are propagated to the efficiency.

Supplementary Note 11. Ground state pulsed M2O magnetic field dependence

As shown in the Figure 4a, we measured the pulsed transduction signal using a magnetic field to tune the ground state spins into resonance with the cavity and then changed the off time between adjacent transduction pulses. In order to determine the spin resonance condition and observe the full spectrum of the polariton splitting, we sweep the magnetic field strength about the ground state spin transition as shown in Figure S12. The nominal pulse sequence of P_o= 550 µW, P_μ= -60 dBm, $\tau_{pulse} = 1$ µs and $\tau_{\text{off}} =$1 ms was used. A horizontal slice at 60 mT in Figure S12 corresponds to the data in Figure 4a at 1 ms off time.

We observed a splitting in the transduction signal when the ground state spins are resonant with the microwave cavity. This phenomenon has previously been observed in Er^{3+}:YSO using a loop-gap microwave resonator [11].We also observed that the transducer efficiency is greater at higher magnetic fields (i.e. 62-64 mT) compared to lower magnetic fields (i.e. 57-59 mT). This asymmetry may be due to hyperfine transitions at the lower magnetic field values that absorb microwave photons and lower the efficiency.

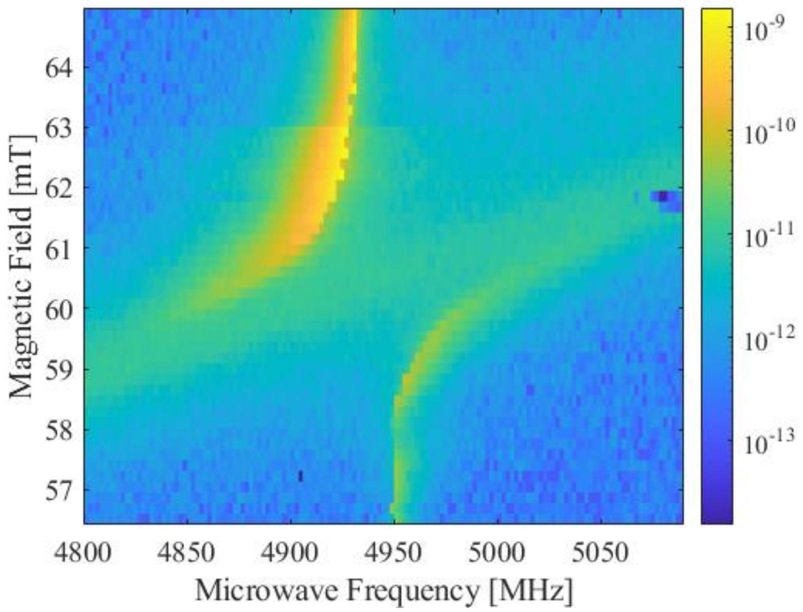

Figure S12: Pulsed transduction efficiency using the ground state spins as a function of the magnetic field.

Supplementary Note 12. Continuous-wave spin optical heating

The temperature of the spins that couple to the microwave cavity as measured during CW optical coupling to the optical device. The spin temperature was inferred by measuring the coupling strength between the spin ensemble and the microwave resonator, which scales as:

$$g_{\mu,\mathrm{tot}} \propto \sqrt{\Delta N} = \sqrt{\frac{1-e^{-\hbar\omega/k_b T}}{1+e^{-\hbar\omega/k_b T}}}. \tag{15}$$

Here, $\Delta N = \sum_k n_{1,k} - n_{2,k}$ is the population difference between the ground state spin levels. The coupling spectrum at different optical powers and the spin ensemble temperature vs optical power is shown in Figure S13. Under CW illumination, the spins reach a temperature 1 K for 30 µW. We assume that under no incident light, the spins are in equilibrium with the mixing chamber stage (i.e. T=35 mK). We attribute the dominant heating source to be light that scatters off the grating coupler and not light within the optical resonator itself. This is based on a measurement where we moved the optical excitation off the grating coupler and measured a similar spin temperature. More efficient optical coupling from fiber can reduce the optical heating. The average power can be reduced by using optical pulses, as shown in Fig. 4. We plot both data sets together in Fig. S13 (d). The two data sets from CW and pulsed measurements indicate the spin temperature has a polynomial dependence on the optical power ($T \sim P_o^{0.3}$).

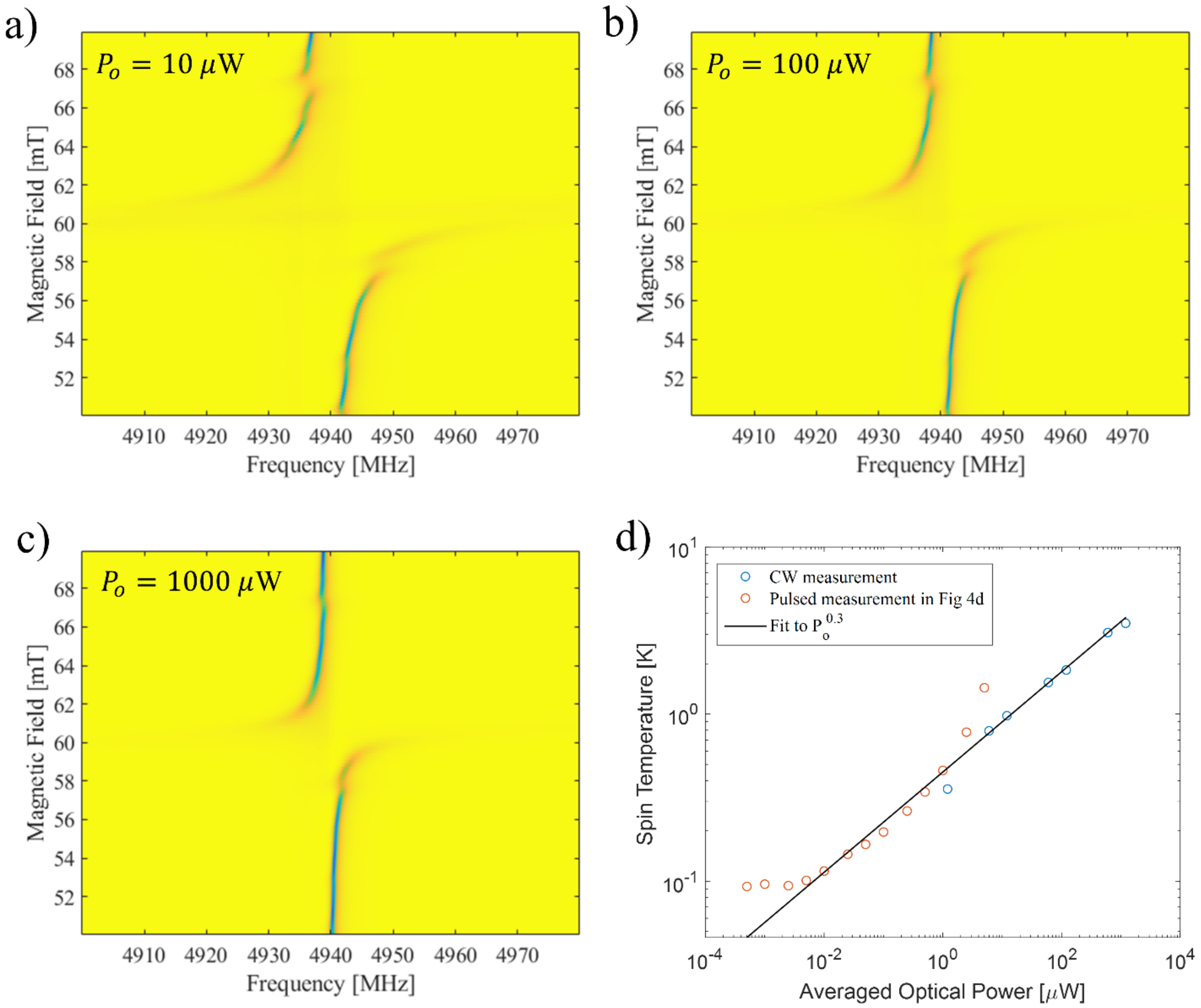

Figure S13: Spin coupling and temperature vs optical power. a-c) The spin-cavity coupling spectrum vs magnetic field for different optical powers. d) The temperature of the spins coupled to the microwave resonator as a function of optical power. Blue circles label the CW measurements and red circles label the pulsed measurement in Fig. 4d.

Supplementary Note 13. Microwave resonator noise theory

To measure the thermal noise of the microwave resonator during the transduction process, we measured the noise that radiates from the microwave resonator into the coupling waveguide through a low noise microwave readout line [12,13].

Our system consists of a microwave resonator that is coupled to its environment through the intrinsic cavity decay rate and to a co-planar waveguide via the coupling cavity decay rate as shown in the inset of Figure 4e. One end of the co-planar waveguide is connected to our microwave amplification readout chain consisting of a low noise HEMT and a room-temperature microwave amplifier for detection. The

other end of the co-planar waveguide is connected to the input microwave line, which is thermally-lagged to the mixing chamber stage with a microwave attenuator. The output thermal noise spectrum from the transducer coupled to the co-planar waveguide is:

$$N_{\mathrm{out}}(\Delta\omega) = \tau(\Delta\omega)\left(N_{\mathrm{env}} - N_{\mathrm{wg}}\right) + N_{\mathrm{wg}} + N_{\mathrm{add}} + 0.5 \quad (16)$$

where $\tau(\Delta\omega) = \frac{2\kappa_{\mu,i}\kappa_{\mu,\mathrm{in}}}{\left(\kappa_{\mu,i}+\kappa_{\mu,\mathrm{in}}\right)^2+4\Delta\omega^2}$ and $N_{out} = P_{\mathrm{out}}/(G \cdot \hbar\omega \cdot \mathrm{BW})$ relates the detected power level to photon number. $\Delta\omega$ is the microwave cavity detuning. N_{env} and N_{wg} are the thermal occupations of the environment the resonator is coupled with and the coupling waveguide, respectively. P_{out} is the output noise photon level after the amplification chain, N_{add} is the added noise from the amplification chain and G is the gain of the microwave amplification chain.

We note that the resonator is coupled to a co-planar waveguide and can radiate into both propagation directions of the waveguide, but we only detect one direction (i.e. $\kappa_{\mu,\mathrm{in}}$ accounts for the resonator coupling to both propagation directions).

Once the N_{wg} and N_{env} have been determined by fitting the noise spectrum, we can calculate the temperature of the resonator mode, N_{mode}, which is coupled to both baths:

$$N_{mode} = \frac{\kappa_{\mu,in}N_{\mathrm{wg}} + \kappa_{\mu,i}N_{\mathrm{env}}}{\kappa_{\mu,in} + \kappa_{\mu,i}} \quad (17)$$

<u>Microwave resonator noise calibration</u>

To calibrate the readout line gain, G, and added noise, N_{add}, a heater was added to the mixing chamber plate and we detected the output microwave power on the spectrum analyzer or digitizer.

The gain and added noise are determined by fitting the noise power to [12]:

$$GN_{\mathrm{out}} = \frac{P_{\mathrm{out}}}{\hbar\omega\mathrm{BW}} = G[\frac{1}{2}\coth\left(\frac{\hbar\omega}{2k_bT}\right) + N_{\mathrm{add}}] \quad (18)$$

where P_{out} is the detected power on the spectrum analyzer or digitizer, N_{out} is the output noise photon number (referenced before the amplifier chain), BW is the detection bandwidth of the detector, G is the gain of the detection setup, T is the temperature and N_{add} is the added noise of the detection setup. A calibration for the detection setup at a frequency of 4.933 GHz using the digitizer is shown in Figure S14, where we fit to G = 90.4$\pm$0.03 dB and N_{add} = 6.84$\pm$0.05 [$s^{-1}Hz^{-1}$]. A similar value for N_{add} was measured when using the spectrum analyzer for detection. The gain and added noise have weak frequency dependence, so they are calibrated for all frequencies in the spectrum.

We note that N_{add} may contain additional noise corresponding to thermal photons in the microwave waveguide when the fridge is at the base temperature (35 mK). There are 40 dB in microwave attenuators on the input coaxial cable to attenuate thermal noise from warmer stages (see Figure S7), which may be insufficient to ensure the waveguide is in thermal equilibrium with the mixing chamber stage. Additional attenuators would require us to send in higher microwave power at the input for some

transduction measurements, which we wanted to avoid. Therefore, in our measurements N_{wg}, N_{env} and N_{mode} correspond to the thermal occupations induced from the optical light within the transducer. The thermal noise of the waveguide introduced from the warmer stages in the fridge can be attenuated using additional attenuators or filtering and is a characterization of the set-up and not the transducer itself.

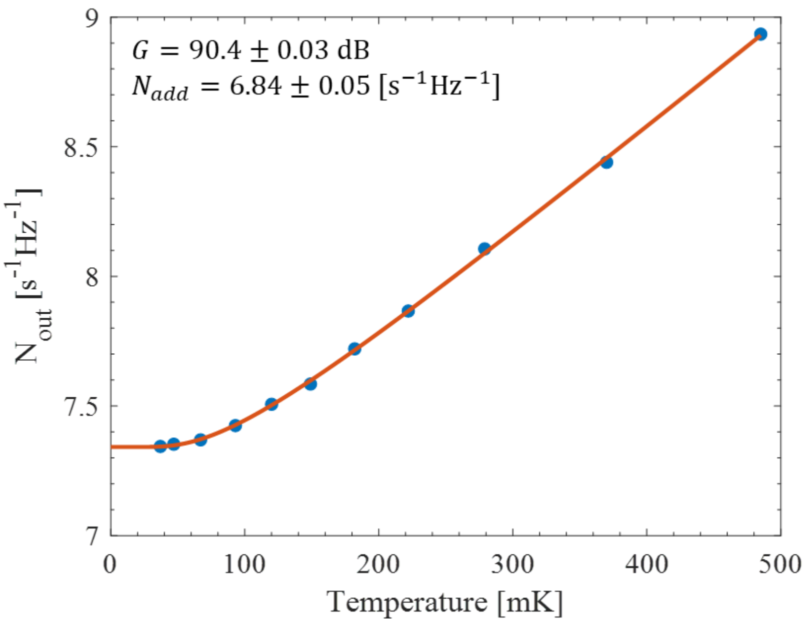

Figure S14: Microwave output amplification chain gain and added noise for the digitizer detection setup.

Supplementary Note 14. Microwave resonator noise – CW optical illumination

The thermal noise of the microwave resonator induced by optical photons was first measured under CW optical illumination at different optical power. The noise spectrum is shown in Figure S15. As we increase the optical power, the microwave resonator shifts to higher frequencies, which we attribute to a decrease in the dispersive shift of the microwave cavity coupling to the ground state spin as the temperature increases.

In CW mode, N_{mode} remains below 0.5 quanta up to P_o=10 µW, while it reaches up to ~3.3 quanta as the optical power reaches P_o=550 µW. The thermal occupancies have a power law dependence N_{wg}~$P_o^{0.53}$, N_{env}~$P_o^{0.34}$ and N_{mode}~$P_o^{0.44}$. The waveguide power law dependence is very similar to the result from Hease et al. [12] who measure N_{wg}~$P_o^{0.55}$ for their system.

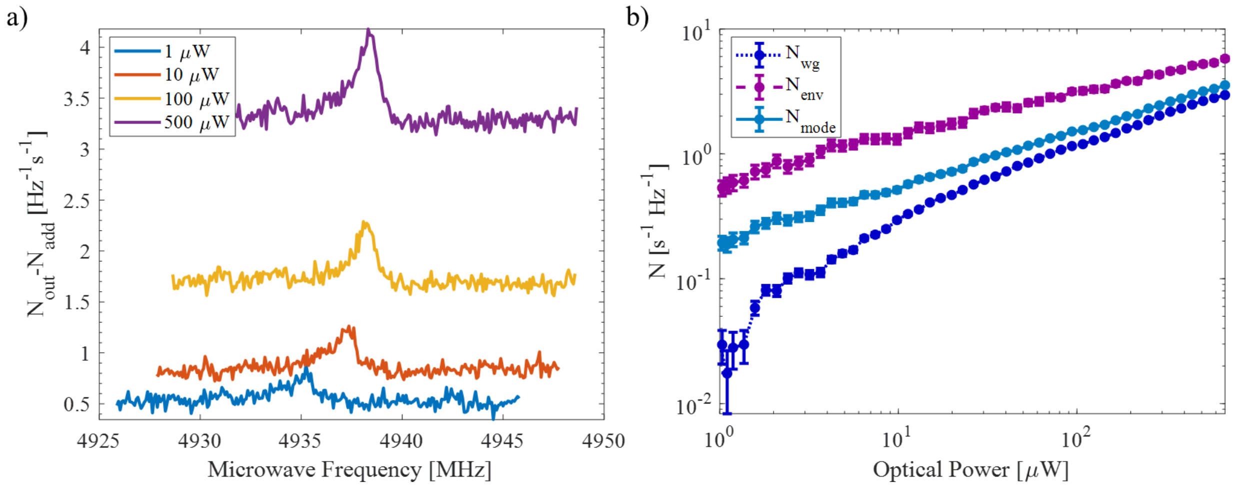

Figure S15: Microwave resonator noise under CW optical illumination. A) Microwave noise spectrum of the device under different optical input power. B) Thermal occupancy for the microwave waveguide, N_{wg}, the resonator environment, N_{env} and the resonator mode, N_{mode} as a function of the optical power. The error bars correspond to the 95% confidence interval of the fitting.

Supplementary Note 15. Microwave resonator noise – pulsed optical illumination

To reduce the average optical power to the transducer, we switched the transducer to pulsed operation. Here, we measured the microwave resonator noise induced by the optical pulses as a function of the parameters for the pulse sequence. The noise as a function of the off time was shown in the Figure 4e, so we will consider the pulse length and optical pump power here.

First, we measured the resonator noise as a function of the pulse length (Figure S16). This was done for P_o= 550 μW and τ_{off} = 10 ms. The thermal occupancies have a power law dependence of N_{wg}~$\tau_{\mathrm{pulse}}{}^{0.65}$, N_{env}~$\tau_{\mathrm{pulse}}{}^{0.37}$ and N_{mode}~ $\tau_{\mathrm{pulse}}{}^{0.4}$. Here we were limited to pulse lengths greater than 10 μs, but we could expect N_{mode} to be ~2-3x smaller for 1 μs pulses (i.e. the pulse length used for the transduction measurements) based on the power law dependence. N_{mode} has similar, but inverse, power law dependencies for τ_{pulse} and τ_{off} (i.e. for τ_{off}<20 ms), which indicates that the thermal occupancy of the mode scales roughly as the average power for the pulse sequences with a duty cycle greater than 0.1% for this pulse sequence.

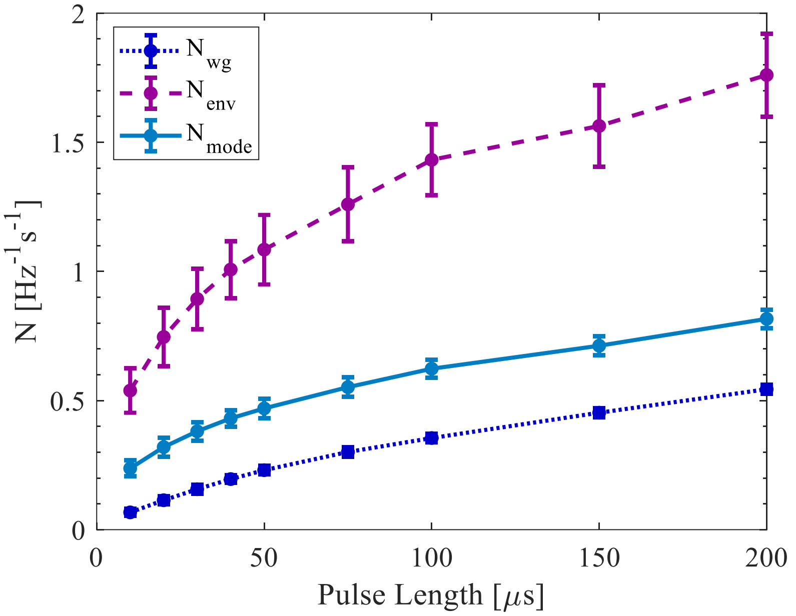

Figure S16: Microwave resonator noise as a function of the optical pulse length. The error bars correspond to the 95% confidence interval of the fitting.

We also measured the resonator noise as a function of the optical pulse power (Figure S17). This was done for τ_{pulse}= 20 µs and τ_{off}= 1 ms. The thermal occupancies have a power law dependence $N_{\text{wg}}{\sim}P_o{}^{0.69}$, $N_{\text{env}}{\sim}P_o{}^{0.58}$ and $N_{\text{mode}}{\sim}P_o{}^{0.55}$ in the pulsed operation, which differ from the continuous wave operation. The average power is 50x smaller compared to the CW experiment (i.e. 2% duty cycle), which may attribute to the different power law scaling. We note that Fu et al. [14] also observed an increased power law dependence for their pulsed measurements compared to their CW measurements when they swept the optical power. The optical light induces <0.1 quanta of noise in the microwave resonator for optical power less than 20 µW.

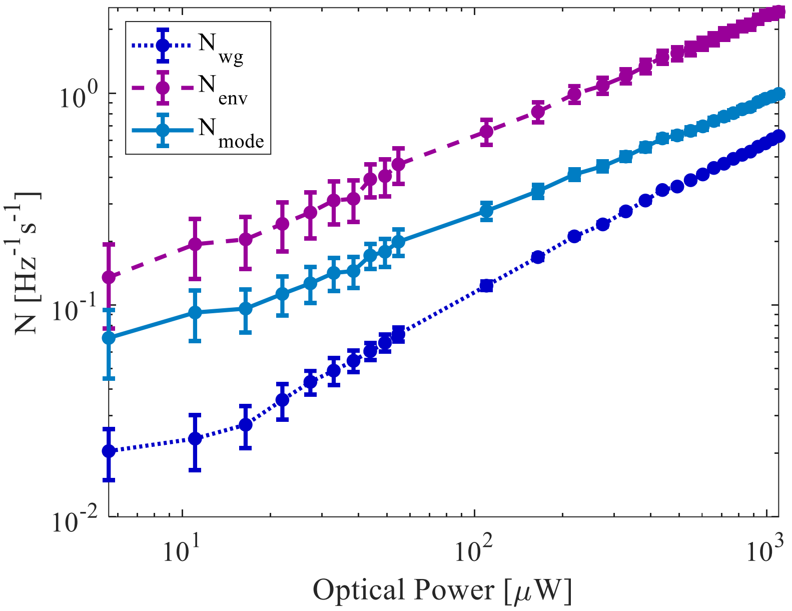

Figure S17: Microwave resonator noise as a function of the applied optical power. The error bars correspond to the 95% confidence interval of the fitting.

Supplementary Note 16. Photoluminescence noise

One noise source specific for transduction with atomic systems is the addition of photoluminescence noise photons during our transduction pulse. That is, the optical pump can excite ions to the optically excited state and when the ions decay spontaneously to the ground state, the emitted photon can act as noise at the transducer optical output.

We quantify this noise in Figure S18, where we measure the detected noise photons per pulse as a function of the off time for our pulsed transduction measurements with spectral filtering and single photon detection. This is done for 1 µs long optical pulses and 550 µW of optical power. We measure the noise photons down to ~$4*10^{-5}$ photons per pulse in the transduction pulse window when the off time is longer than the optical lifetime of 3 ms. The red dashed line is the detection noise floor of our setup which includes SNSPD background counts and laser leakage from the filtering. After accounting for detection losses, the photoluminescence noise referred to the output of the optical device is 15 dB higher (or ~10^{-3} photons per pulse).

We also determined that these noise photons correspond to photoluminescence from ions in the bulk of the crystal (i.e. not from our transducer). This was revealed by moving the optical excitation off the grating coupler and measuring the same amount of photoluminescence. Therefore, this is not fundamental to a REI transducer and will not be a problem if we remove those ions in future devices. Nonetheless, the noise photon rate is already quite small due to the high extinction of the filtering that removes most of the photoluminescence noise and the time domain filtering associated with short transduction pulses (i.e. pulse length is much shorter than the optical lifetime).

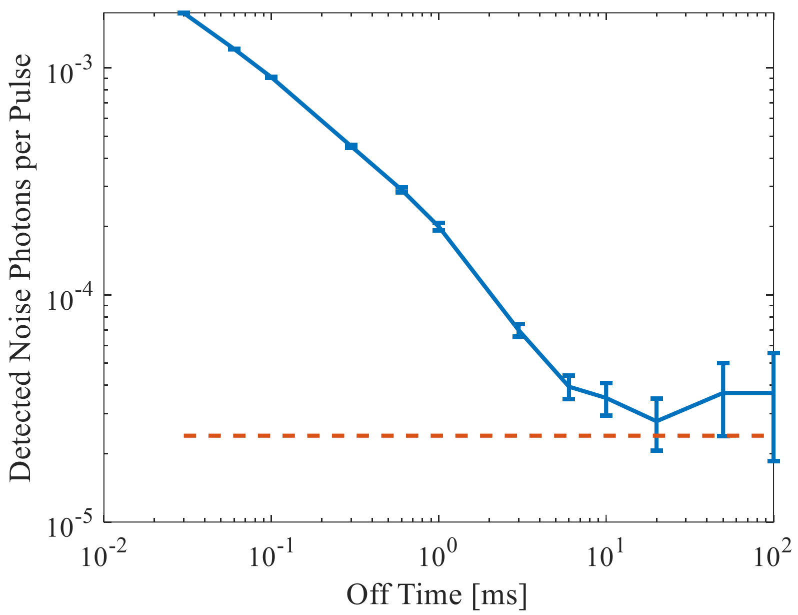

Figure S18: Detected photoluminescence noise per transduction pulse as a function of the off time between adjacent pulses. The red dashed line is the detection noise floor. Error bars correspond to $\sqrt{\text{counts}}$ measured on the SNSPD.

Supplementary Note 17. Path to high efficiency and low noise transduction

The main improvements needed for a future device include increasing the transduction efficiency and achieving low noise operation with higher duty-cycle transduction pulses. Both efficiency and noise performance can be improved at the same time by more efficient use of the optical photons. This could be achieved with a significantly over-coupled optical cavity and on/off chip coupling with near unity efficiency. Under such conditions, lower optical power would yield reduced noise while maintaining similar optical Rabi frequencies, and lead to higher efficiency through better collection of the transduced photons.

Another issue with the current device is the parasitic spins. This included the even isotope erbium spins that were within the microwave cavity but not the optical cavity that impacted the ground state transduction efficiency and the ^{167}Er spins that impacted the excited state transduction. The ^{167}Er spins are easier to avoid by moving to an isotopically purified sample. From simulation, the parasitic spins decrease the ground state transduction efficiency by 100x and the excited state efficiency by 3x.

Removing the parasitic even isotope spins can be achieved by moving to a different fabrication platform, where the erbium spins are only within the optical cavity. For example, the optical resonator can be patterned directly out of the $Er^{3+}:YVO_4$ material instead of relying on patterning amorphous silicon and evanescent coupling. This would also increase the number of spins by 14x and the optical pump Rabi frequency by ~3x as the optical mode would be predominately within the $Er^{3+}:YVO_4$ material. Thus,

patterning the optical cavity directly out of $Er^{3+}:YVO_4$ is predicted to increase the transduction efficiency to $\eta_{d,\mathrm{int}} = 10^{-2}$.

There are several possible paths to increase the efficiency further. The microwave coupling can be increased by reducing the parasitic inductance of the microwave resonator. Only 20% of the resonator inductance is confined to the skinny inductive wire. The parasitic inductance of the interdigitated capacitor can be reduced significantly by using a parallel plate capacitor, albeit at the cost of increased fabrication complexity. Removing the parasitic inductance can increase g_μ by $\sqrt{5}$x and the transduction efficiency by 5x.

The optical pump Rabi frequency can be significantly increased by applying it resonantly with a high Q optical cavity mode. The mean intracavity photon number, $\langle n_p \rangle$, of the optical pump is given by $\langle n_p \rangle = \frac{P_{o,\mathrm{in}}}{\hbar\omega_o} \frac{\kappa_{o,\mathrm{in}}}{\kappa_o^2/4 + \delta_{c,o}^2}$, where $P_{o,\mathrm{in}}$ is the input pump optical power. In the case of an optical pump resonant with a cavity mode with a quality factor of 100,000 that is dominated by input coupling losses, the intracavity photon number can be increased by a factor of 20x compared to the current device for the same input optical pump power. The transduction efficiency should increase linearly with the intracavity pump photon number. We note that this would require redesigning the optical cavity such that it includes two cavity modes that are resonant with the pump and transduction signal.

Another ion host system may also offer stronger (i.e. larger dipole moment) and/ or more narrow transitions that can improve the coupling rates. For example, $Yb^{3+}:YVO_4$ offers stronger transitions and narrower transitions, even at 6x lower doping concentration. Using the spectroscopic parameters from [15], $^{171}Yb^{3+}:YVO_4$ can achieve $\alpha = 1.6 \cdot 10^{-8}$, which is a factor of 100x larger than the value for $Er^{3+}:YVO_4$. Also, $^{171}Yb^{3+}:YVO_4$ has improved optical and spin coherence properties compared to $Er^{3+}:YVO_4$, which increases the maximum efficiency achievable. This change, if implemented with the above changes, would result in an increase in efficiency to near unity ($\eta_{d,\mathrm{int}} > 90\%$).”

We note that a large portion of the improvement by using $^{171}Yb^{3+}:YVO_4$ is due to the significantly narrower spin linewidth (130 kHz compared to 65 MHz), which may be difficult to fully maintain due to magnetic field inhomogeneity near the superconducting microwave resonator. Future experiments will determine the spin inhomogeneity near superconducting microwave resonators.

Supplementary References

1. Diniz, I. *et al.* Strongly coupling a cavity to inhomogeneous ensembles of emitters: Potential for long-lived solid-state quantum memories. *Physical Review A* **84,** (2011).
2. Williamson, L. A., Chen, Y.-H. & Longdell, J. J. Magneto-optic modulator with unit quantum efficiency. *Physical Review Letters* **113,** (2014).
3. Barnett, P. S. & Longdell, J. J. Theory of microwave-optical conversion using rare-earth-ion dopants. *Physical Review A* **102,** (2020).
4. Fernandez-Gonzalvo, X., Horvath, S. P., Chen, Y.-H. & Longdell, J. J. Cavity-enhanced Raman heterodyne spectroscopy in Er^{3+}:Y_2SiO_5 for microwave to optical signal conversion. *Physical Reviews A* **100**, 033807 (2019).
5. Bienfait, A. *et al.* Reaching the quantum limit of sensitivity in electron spin resonance. *Nature Nanotechnology* **11,** 253–257 (2015).
6. Eichler, C., Sigillito, A. J., Lyon, S. A. & Petta, J. R. Electron spin resonance at the level of 10^4 spins using low impedance superconducting resonators. *Physical Review Letters* **118,** (2017).
7. Xie, T. *et al.* Characterization of Er^{3+}:YVO_4 for microwave to optical transduction. *Physical Review B* **104,** (2021).
8. Afzelius, M. *et al.* Efficient optical pumping of Zeeman spin levels in Nd^{3+}:YVO_4. *Journal of Luminescence* **130,** 1566–1571 (2010).
9. Ranon, U. Paramagnetic resonance of Nd^{3+}, Dy^{3+}, Er^{3+} and Yb^{3+} in YVO_4. *Physics Letters A* **28,** 228–229 (1968).
10. Abragam, A. & Bleaney, B. *Electron paramagnetic resonance of transition ions*. (Clarendon Press, 1970).
11. King, G. G., Barnett, P. S., Bartholomew, J. G., Faraon, A. & Longdell, J. J. Probing strong coupling between a microwave cavity and a spin ensemble with Raman Heterodyne spectroscopy. *Physical Review B* **103,** (2021).
12. Hease, W. *et al.* Bidirectional electro-optic wavelength conversion in the quantum ground state. *PRX Quantum* **1,** (2020).
13. Xu, M. *et al.* Radiative cooling of a superconducting resonator. *Physical Review Letters* **124,** (2020).
14. Fu, W. *et al.* Cavity Electro-optic circuit for microwave-to-optical conversion in the Quantum Ground State. *Physical Review A* **103,** (2021).
15. Bartholomew, J.G. *et al.* On-chip coherent microwave-to-optical transduction mediated by ytterbium in YVO_4. *Nature Communications* **11,** 3266 (2020).
